# A biomarker analysis of plasma RIME and PD-L1 for stratifying first-line immunochemotherapy outcomes in advanced ESCC

**DOI:** 10.1016/j.isci.2026.117995

**Published:** 2026-10-23

**Authors:** Jia Liu, Rui-Qi Ren, Ye He, Qi-Hua Zhang, Xiao-Min Zhang, Xiao-Jing Luo, Yu-Zhe Cao, Qi-Nian Wu, Hui-Yan Luo

**Affiliations:** 1 State Key Laboratory of Oncology in South China, Sun Yat-sen University Cancer Center, 651 Dongfeng East Road, Guangzhou 510060, P.R. China; 2 Biotechnology, School of Laboratory Medicine and Biotechnology, Southern Medical University, Guangzhou, Guangdong 510515, China

**Keywords:** ESCC, PD-1 inhibitor, PD-L1, biomarker, RIME

## Abstract

Clinical benefit from first-line PD-1 inhibitor plus chemotherapy in advanced ESCC remains heterogeneous, and PD-L1 alone provides limited stratification. We evaluated plasma lncRNA RIME as a potential biomarker for stratifying first-line treatment outcomes in a multicenter cohort of patients with advanced ESCC. In the chemotherapy plus PD-1 inhibitor group, RIME-low was associated with higher ORR and better PFS/OS, whereas RIME-high was associated with poorer outcomes. Joint RIME/PD-L1 assessment further distinguished clinical benefit. Nested-model analyses showed that RIME provided non-redundant outcome information beyond PD-L1, and treatment-by-RIME interactions supported an exploratory predictive signal for ORR and PFS. Overall, plasma RIME was associated with outcome stratification and represents a potential predictive biomarker for immunochemotherapy benefit in ESCC.

## Highlights

• PD-L1 alone has limited value in stratifying immunochemotherapy benefit in ESCC
• Plasma RIME stratifies outcomes: RIME-low has higher ORR and better PFS/OS
• Joint RIME/PD-L1 assessment further distinguishes clinical benefit
• RIME-low/PD-L1-high shows the best outcomes; RIME-high/PD-L1-high the worst

## Graphical abstract

**Figure:**
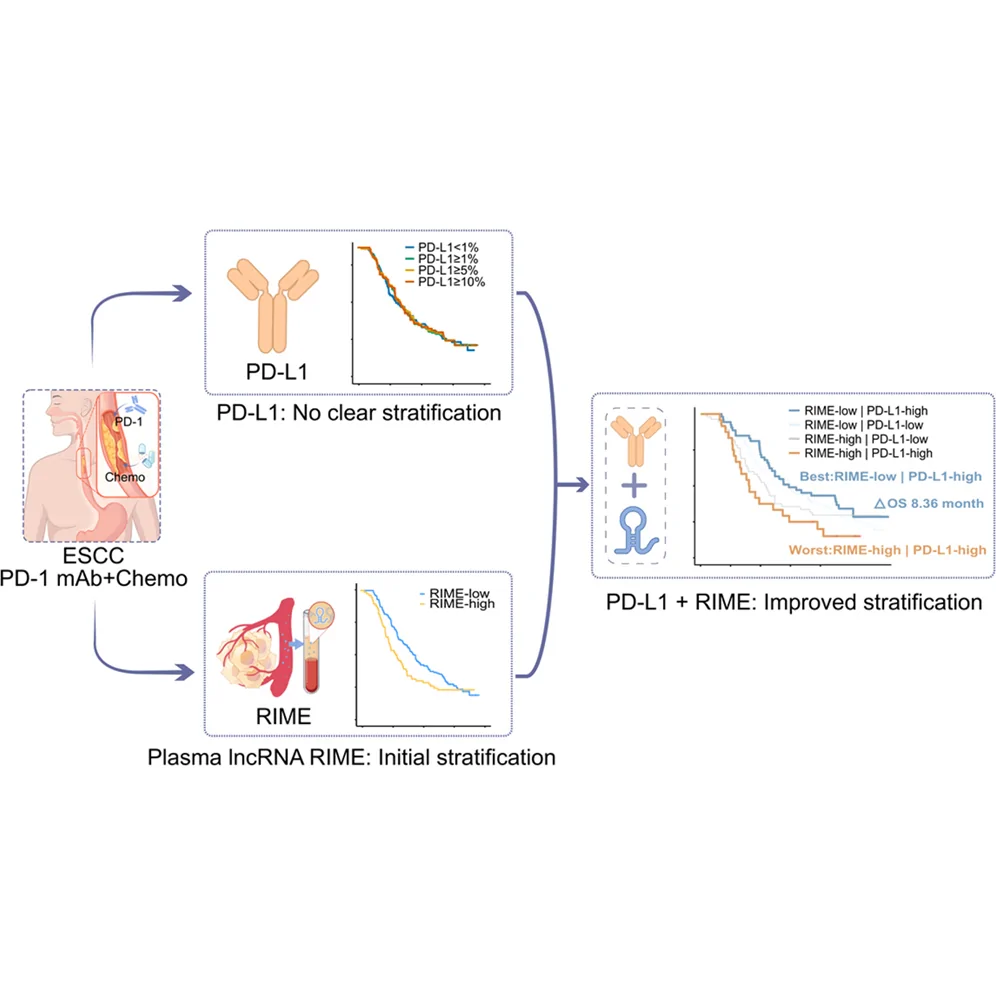

## Teaser

Oncology; Therapeutics

## Introduction

Esophageal cancer is one of the leading causes of cancer-related death worldwide and carries a particularly heavy burden in East Asia.^1^ Despite continuous advances in treatment strategies, the overall prognosis of advanced/metastatic esophageal squamous cell carcinoma (ESCC) remains poor, and the long-term benefit of systemic therapy is limited.^1^^,^^2^^,^^3^ Multiple phase III randomized controlled trials have shown that first-line PD-1 inhibitor plus platinum-based chemotherapy improves objective response rates and survival outcomes and has become an important standard-of-care strategy.^4^^,^^5^^,^^6^^,^^7^ Nevertheless, even in the setting of combined immunochemotherapy, clinical outcomes remain markedly heterogeneous: some patients achieve durable responses, whereas others still experience early progression or limited benefit.^4^^,^^8^ This observation suggests that current stratification markers remain insufficient to fully explain differences in outcomes under combined treatment.

Among currently available immunotherapy-related markers, PD-L1 expression remains one of the most commonly used stratification factors and has been prespecified in multiple clinical trials.^8^^,^^9^ However, the predictive value of PD-L1 remains controversial in ESCC.^10^^,^^11^ Some studies suggest that higher PD-L1 expression is associated with more evident survival benefit,^5^^,^^12^ whereas results in lower-threshold-defined strata have been inconsistent across studies. In addition, differences in antibodies, scoring systems (such as CPS, TPS, or TAP), and cutoff definitions further limit cross-study comparison and clinical interpretation. Notably, a recent meta-analysis incorporating data presented at the September 2024 FDA Oncologic Drugs Advisory Committee meeting reported no significant OS benefit from PD-1/PD-L1 inhibitors in PD-L1-negative ESCC,^13^ whereas a post hoc TPS-stratified analysis of JUPITER-06 suggested that clinical benefit from PD-1 inhibitor plus chemotherapy could be observed regardless of TPS level.^14^ Overall, current evidence supports viewing PD-L1 as a clinically meaningful but insufficient baseline stratification marker, rather than a decisive biomarker capable of independently and stably distinguishing groups with different clinical benefit.^10^ Meanwhile, many emerging biomarker strategies for treatment-response prediction rely on genomic or transcriptomic profiling using high-throughput platforms.^15^^,^^16^^,^^17^ Although biologically informative, these approaches are often costly, technically demanding, and less convenient for routine clinical application.^16^^,^^18^ Therefore, there remains a need for practical and accessible biomarkers that can more readily distinguish patients with differing levels of clinical benefit and better inform treatment decisions in first-line immunochemotherapy.

Our previous study demonstrated that RIME promotes immune escape in ESCC by increasing H3K4me3 enrichment at the PD-L1 and IDO-1 promoters, thereby upregulating PD-L1 and IDO-1 expression, reducing intratumoral T cell infiltration and activity, and contributing to immunotherapy resistance in ESCC.^19^ On this basis, we examined the association of pretreatment plasma RIME expression with clinical outcomes using specimens and data from a multicenter cohort of patients with advanced ESCC. Plasma lncRNA RIME expression was measured in pretreatment plasma specimens. Patients were classified into high- and low-expression groups using an exploratory, analysis-derived threshold, and ORR, PFS, and OS were evaluated to determine whether plasma lncRNA RIME could identify groups with markedly different clinical benefit and whether it was associated with adverse events. In addition, subgroup analyses were performed within the chemotherapy plus PD-1 inhibitor group using nested PD-L1 threshold definitions (<1%, ≥1%, ≥5%, and ≥10%) to evaluate the clinical benefit discrimination achieved by the combination of RIME and PD-L1. This study aimed to define the value of plasma lncRNA RIME in distinguishing clinical benefit in patients treated with PD-1 inhibitor plus chemotherapy and to assess whether its joint use with PD-L1 could more precisely distinguish patients with different levels of clinical benefit.

## Results

### Baseline characteristics by RIME status

A total of 447 patients with available baseline plasma lncRNA RIME measurements were included in the analysis. Based on measured baseline RIME values, 79 of 218 patients (36.2%) in the chemotherapy group and 81 of 229 patients (35.4%) in the chemotherapy plus PD-1 inhibitor group were classified as RIME-high, while the remaining 139 (63.8%) and 148 (64.6%) patients, respectively, were classified as RIME-low (Table 1). Plasma lncRNA RIME expression levels were derived from RNA expression data, normalized to the polyA spike-in control to correct for differences in RNA input and assay efficiency across samples. Averaged technical replicate values were log-transformed and used as the continuous expression variable for subsequent analyses (see STAR Methods). To derive a clinically interpretable binary grouping, we performed time-dependent ROC analysis anchored to 6-month progression-free status (PFS 6 months) for descriptive assessment and used maximally selected rank statistics based on PFS time and event status to determine the cutoff, thereby classifying patients into RIME-high and RIME-low groups. Baseline characteristics were generally comparable between groups, including age, sex, ECOG performance status, disease status (locally advanced or distant metastatic disease), number of metastatic sites, liver metastasis, prior chemoradiotherapy, and PD-L1 subgroup distribution (Table 1).

**Table 1:** Baseline characteristics by RIME expression group

Characteristic	Chemo, RIME-low (*N* = 139)	Chemo, RIME-high (*N* = 79)	Chemo+PD-1mAb, RIME-low (*N* = 148)	Chemo+PD-1mAb, RIME-high (*N* = 81)
Age, years	61.2 (7.0)	62.9 (6.7)	60.7 (6.4)	61.0 (7.2)
**Sex**				
Male	129 (92.8)	66 (83.5)	132 (89.2)	69 (85.2)
Female	10 (7.2)	13 (16.5)	16 (10.8)	12 (14.8)
Missing	0 (0.0)	0 (0.0)	0 (0.0)	0 (0.0)
**ECOG**				
0	32 (23.0)	15 (19.0)	29 (19.6)	23 (28.4)
1	107 (77.0)	64 (81.0)	119 (80.4)	58 (71.6)
Missing	0 (0.0)	0 (0.0)	0 (0.0)	0 (0.0)
**Disease status**				
Locally advanced	29 (20.9)	10 (12.7)	34 (23.0)	14 (17.3)
Distant metastasis	110 (79.1)	69 (87.3)	114 (77.0)	67 (82.7)
Missing	0 (0.0)	0 (0.0)	0 (0.0)	0 (0.0)
**Liver metastasis**				
No	110 (79.1)	57 (72.2)	116 (78.4)	59 (72.8)
Yes	29 (20.9)	22 (27.8)	32 (21.6)	22 (27.2)
Missing	0 (0.0)	0 (0.0)	0 (0.0)	0 (0.0)
**PD-L1 (TPS)**				
<1%	61 (43.9)	32 (40.5)	66 (44.6)	35 (43.2)
≥1%	74 (53.2)	46 (58.2)	80 (54.1)	44 (54.3)
Missing	4 (2.9)	1 (1.3)	2 (1.4)	2 (2.5)
**Prior chemoradiotherapy**				
No	131 (94.2)	75 (94.9)	135 (91.2)	78 (96.3)
Yes	8 (5.8)	4 (5.1)	13 (8.8)	3 (3.7)
Missing	0 (0.0)	0 (0.0)	0 (0.0)	0 (0.0)
**Metastatic sites**				
1	79 (56.8)	38 (48.1)	70 (47.3)	39 (48.1)
≥2	60 (43.2)	41 (51.9)	78 (52.7)	42 (51.9)
Missing	0 (0.0)	0 (0.0)	0 (0.0)	0 (0.0)
Baseline polyA-normalized log RIME expression	−3.9 (3.4)	6.3 (4.2)	−3.7 (3.4)	6.1 (3.9)

Data are presented as mean (SD) for continuous variables and n (%) for categorical variables. Percentages are based on column totals. RIME grouping was determined using an exploratory cutoff derived by maximally selected rank statistics based on PFS time and event status. RNA expression data were processed by averaging Ct values across technical replicates, followed by normalization to the polyA spike-in control using the 2^−ΔCt method and subsequent log transformation for analysis.

### Efficacy and survival outcomes across PD-L1-defined subgroups in the chemotherapy plus PD-1 inhibitor group

To evaluate the discrimination performance of PD-L1 alone within the chemotherapy plus PD-1 inhibitor group, we performed separate threshold-specific comparisons using PD-L1 <1% as the reference and the nested PD-L1-positive definitions of ≥1%, ≥5%, and ≥10%. The three positive populations overlapped and were not treated as mutually exclusive categories. Descriptive Kaplan-Meier curves showed limited separation in OS across the threshold-defined populations (Figure 1A), with median OS values of 16.59 months for PD-L1 <1%, 16.30 months for PD-L1 ≥1%, 17.28 months for PD-L1 ≥5%, and 15.64 months for PD-L1 ≥10%. In separate Cox models, neither PFS nor OS differed significantly between PD-L1 <1% and any of the nested positive-threshold populations, with HRs generally close to 1 (Figure 1B). PFS curves likewise showed limited separation (Figure 1C), with median PFS values of 7.72 months for PD-L1 <1% and 7.00 months for each of the PD-L1 ≥1%, ≥5%, and ≥10% populations. ORR estimates showed no consistent gradient across increasing PD-L1 thresholds (Figure 1D). Taken together, these descriptive and threshold-specific comparisons indicate that PD-L1 alone had limited ability to distinguish clinical benefit in the chemotherapy plus PD-1 inhibitor group.

**Figure 1:**
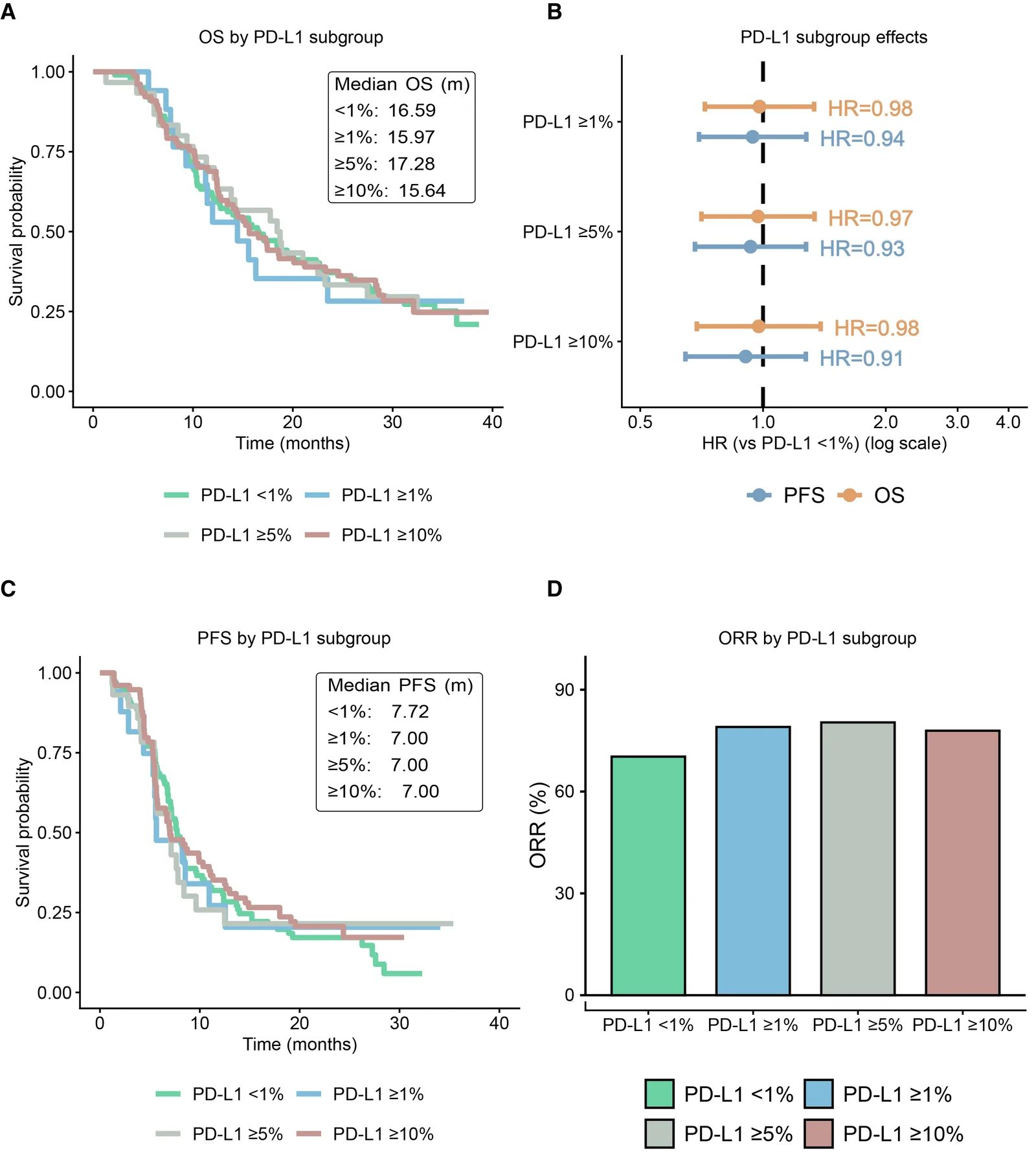
Efficacy and survival outcomes across PD-L1-defined subgroups in the chemotherapy plus PD-1 inhibitor group PD-L1 threshold-defined groups included PD-L1 <1% (*n* = 101), ≥1% (*n* = 124), ≥5% (*n* = 107), and ≥10% (*n* = 77). (A) Kaplan-Meier estimates of overall survival (OS) by PD-L1 threshold-defined subgroup. (B) Hazard ratios (HRs) for progression-free survival (PFS) and OS from separate univariable Cox proportional hazards models comparing each PD-L1-positive threshold with the PD-L1 <1% subgroup; points indicate HRs and horizontal lines indicate 95% confidence intervals. (C) Kaplan-Meier estimates of PFS by PD-L1 threshold-defined subgroup. (D) Observed objective response rates (ORRs) by PD-L1 threshold-defined subgroup. Kaplan–Meier curves are descriptive. n represents individual patients.

### Objective response by baseline RIME status across treatment groups and PD-L1-defined subgroups

Among response-evaluable patients, ORR was similar between the RIME-low and RIME-high groups in the chemotherapy group, at 70.59% (96/136) and 65.79% (50/76), respectively (Figure 2A). By contrast, in the overall chemotherapy plus PD-1 inhibitor group, ORR was markedly higher in the RIME-low group than in the RIME-high group, at 84.25% (123/146) and 61.54% (48/78), respectively (Figure 2B). Further stratification within the chemotherapy plus PD-1 inhibitor group by PD-L1 thresholds showed pronounced ORR differences between RIME-low and RIME-high in the PD-L1 ≥1%, ≥5%, and ≥10% strata, with nominal P values below 0.05. In the PD-L1 ≥1% stratum (Figure 2D), ORRs were 91.14% (72/79) and 60.47% (26/43) in the RIME-low and RIME-high groups, respectively; in the PD-L1 ≥5% stratum (Figure 2E), the corresponding values were 91.30% (63/69) and 63.89% (23/36); and in the PD-L1 ≥10% stratum (Figure 2F), they were 88.68% (47/53) and 54.17% (13/24). In contrast, in the PD-L1 <1% stratum (Figure 2C), ORRs were 76.92% (50/65) and 63.64% (21/33), showing the same directional trend but without statistical significance. Overall, these exploratory findings suggest that RIME-low is associated with higher ORR in the chemotherapy plus PD-1 inhibitor group and that joint evaluation with PD-L1 may provide a clearer descriptive presentation of ORR differences across patient groups.

**Figure 2:**
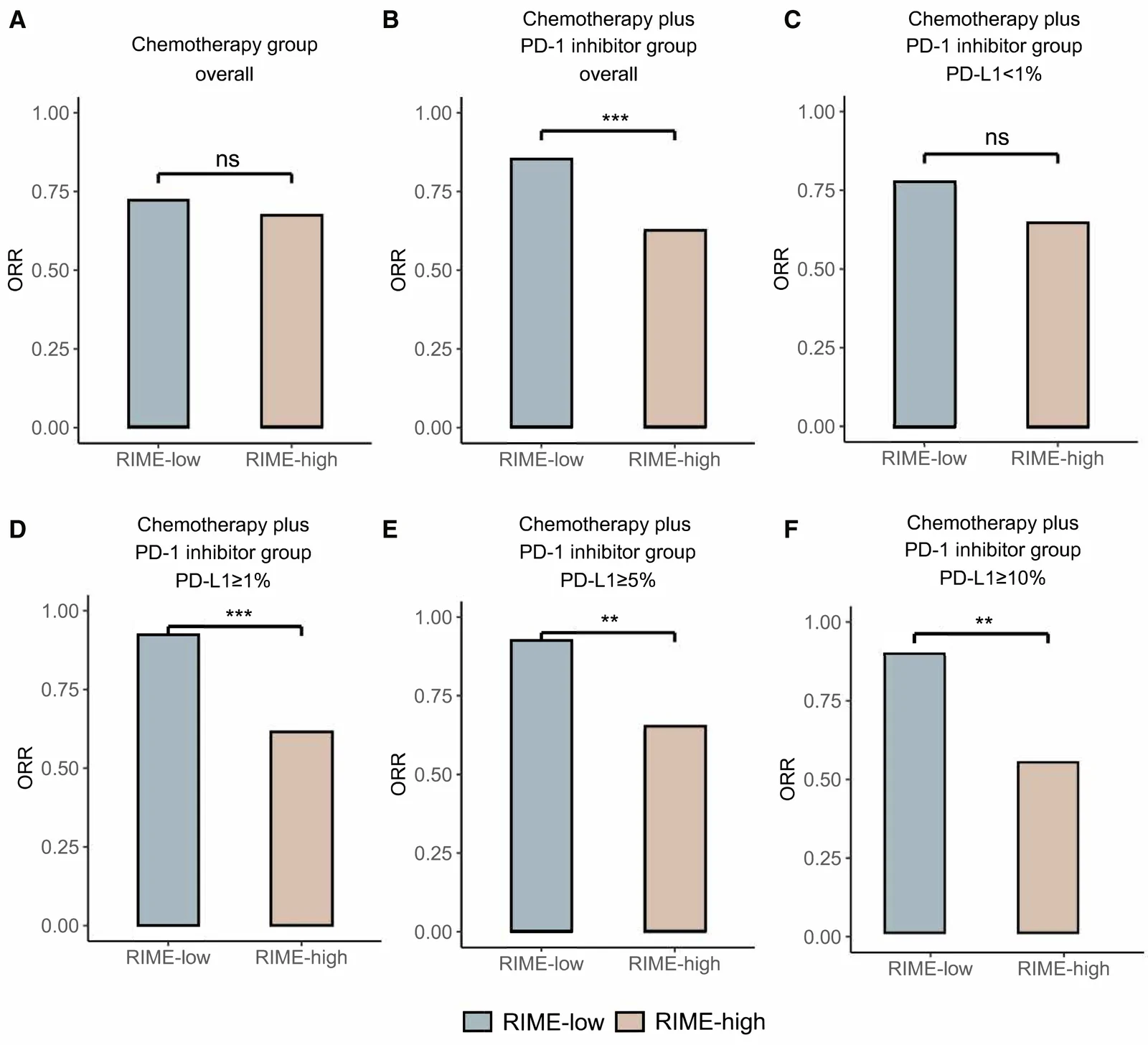
Objective response by baseline RIME status across treatment groups and PD-L1-defined subgroups Bars show observed ORRs among response-evaluable patients; n represents response-evaluable patients. Patients with investigator-assessed complete response, partial response, stable disease, or progressive disease were considered response-evaluable; Patients without a post-baseline tumor assessment (NA) or with a non-evaluable response (NE) were excluded. Comparisons between RIME-low and RIME-high groups were performed using two-sided Fisher’s exact tests. (A) Chemotherapy group overall: RIME-low, 96/136; RIME-high, 50/76; P = 0.537. (B) Chemotherapy plus PD-1 inhibitor group overall: RIME-low, 123/146; RIME-high, 48/78; P < 0.001. (C) PD-L1 <1% subgroup: RIME-low, 50/65; RIME-high, 21/33; P = 0.231. (D) PD-L1 ≥1% subgroup: RIME-low, 72/79; RIME-high, 26/43; P < 0.001. (E) PD-L1 ≥5% subgroup: RIME-low, 63/69; RIME-high, 23/36; P = 0.001. (F) PD-L1 ≥10% subgroup: RIME-low, 47/53; RIME-high, 13/24; P = 0.002. No error bars are shown because bars represent observed proportions. ns, not significant; ∗∗P < 0.01; ∗∗∗P < 0.001.

### Overall survival by baseline RIME status across treatment groups and PD-L1-defined subgroups

To evaluate the association between plasma lncRNA RIME and overall survival, we compared OS between the RIME-low and RIME-high groups in the chemotherapy group overall, the chemotherapy plus PD-1 inhibitor group overall, and PD-L1-defined strata within the chemotherapy plus PD-1 inhibitor group. Kaplan–Meier analysis showed that, in the chemotherapy group overall, RIME-high was associated with poorer OS (HR=1.41, 95% CI 1.05–1.91; log-rank p=0.023; Figure 3A). In the chemotherapy plus PD-1 inhibitor group overall, the same directional trend was observed, although it did not reach statistical significance (HR=1.32, 95% CI 0.96–1.82; log-rank p=0.083; Figure 3B). Further stratified analyses within the chemotherapy plus PD-1 inhibitor group showed no evident difference between RIME-low and RIME-high in the PD-L1 <1% stratum (HR=1.02, 95% CI 0.62–1.67; log-rank p=0.939; Figure 3C), whereas in the PD-L1 ≥1%, ≥5%, and ≥10% strata, RIME-high showed nominally worse OS, with HRs of 1.74 (95% CI 1.13–2.68; p=0.010), 1.73 (95% CI 1.09–2.75; p=0.020), and 2.01 (95% CI 1.15–3.50; p=0.012), respectively (Figures 3D–3F). Consistently, median OS bar plots showed longer median OS in the RIME-low group than in the RIME-high group in the PD-L1 ≥1%, ≥5%, and ≥10% strata, whereas differences were minimal in the PD-L1 <1% stratum (Figure 3G). Overall, these findings show an association between RIME status and OS in the chemotherapy plus PD-1 inhibitor group, and joint evaluation with PD-L1 allows clearer presentation of OS differences across strata, while discrimination remains limited in the PD-L1 <1% stratum.

**Figure 3:**
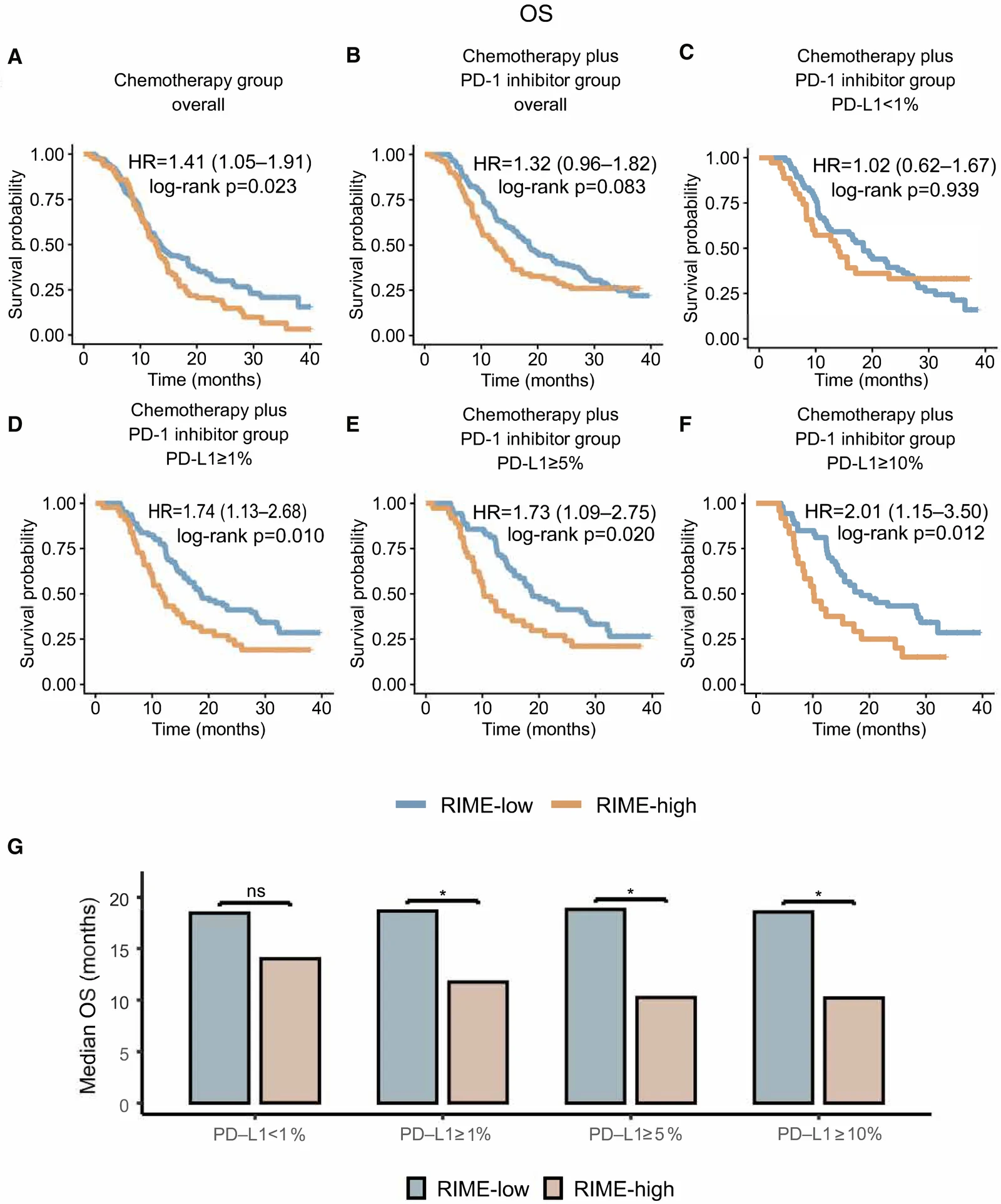
Overall survival by baseline RIME status across treatment groups and PD-L1-defined subgroups Kaplan-Meier curves show OS according to RIME-low versus RIME-high status; *n* represents individual patients. HRs compare RIME-high with RIME-low and were estimated using univariable Cox proportional hazards models; 95% confidence intervals are shown in parentheses. *p* values were calculated using two-sided log rank tests. Tick marks indicate censored observations. (A) Chemotherapy group overall (*n* = 218): HR = 1.41 (95% CI, 1.05–1.91), *p* = 0.023. (B) Chemotherapy plus PD-1 inhibitor group overall (*n* = 229): HR = 1.32 (95% CI, 0.96–1.82), *p* = 0.083. (C) PD-L1 <1% subgroup (*n* = 101): HR = 1.02 (95% CI, 0.62–1.67), *p* = 0.939. (D) PD-L1 ≥1% subgroup (*n* = 124): HR = 1.74 (95% CI, 1.13–2.68), *p* = 0.010. (E) PD-L1 ≥5% subgroup (*n* = 107): HR = 1.73 (95% CI, 1.09–2.75), *p* = 0.020. (F) PD-L1 ≥10% subgroup (*n* = 77): HR = 2.01 (95% CI, 1.15–3.50), *p* = 0.012. (G) Median OS by RIME status across PD-L1-defined subgroups. ns, *p* ≥ 0.05; ∗*p* < 0.05.

### Progression-free survival by baseline RIME status across treatment groups and PD-L1-defined subgroups

To evaluate the association between plasma lncRNA RIME and progression-free survival, we compared PFS between the RIME-low and RIME-high groups in the chemotherapy group overall, the chemotherapy plus PD-1 inhibitor group overall, and PD-L1-defined strata within the chemotherapy plus PD-1 inhibitor group. Kaplan–Meier analysis showed that in the chemotherapy group overall, the association between RIME-high and poorer PFS did not reach statistical significance (HR=1.21, 95% CI 0.89–1.64; log-rank p=0.221; Figure 4A), whereas in the chemotherapy plus PD-1 inhibitor group overall, RIME-high was nominally associated with worse PFS (HR=1.38, 95% CI 1.01–1.89; log-rank p=0.046; Figure 4B). Further stratified analyses within the chemotherapy plus PD-1 inhibitor group showed no evident difference between RIME-low and RIME-high in the PD-L1 <1% stratum (HR=0.93, 95% CI 0.57–1.52; log-rank p=0.771; Figure 4C), whereas in the PD-L1 ≥1% and ≥5% strata, RIME-high showed a nominally higher risk of progression, with HRs of 2.01 (95% CI 1.31–3.08; p=0.001) and 1.83 (95% CI 1.15–2.90; p=0.010), respectively (Figures 4D and 4E). In the PD-L1 ≥10% stratum, the same directional trend was observed but did not reach statistical significance (HR=1.70, 95% CI 0.97–2.98; p=0.063; Figure 4F). Consistently, median PFS was longer in the RIME-low group than in the RIME-high group in the PD-L1 ≥1%, ≥5%, and ≥10% strata, at 8.74 vs 5.49 months, 8.74 vs 5.45 months, and 9.89 vs 5.49 months, respectively; in contrast, the difference was small in the PD-L1 <1% stratum (8.21 vs 7.06 months; Figure 4G). Overall, these findings indicate that plasma lncRNA RIME distinguishes groups with different PFS outcomes in the chemotherapy plus PD-1 inhibitor group; this distinction is clearer in the PD-L1 ≥1% and ≥5% strata, shows a consistent trend in the PD-L1 ≥10% stratum, and remains relatively limited in the PD-L1 <1% stratum.

**Figure 4:**
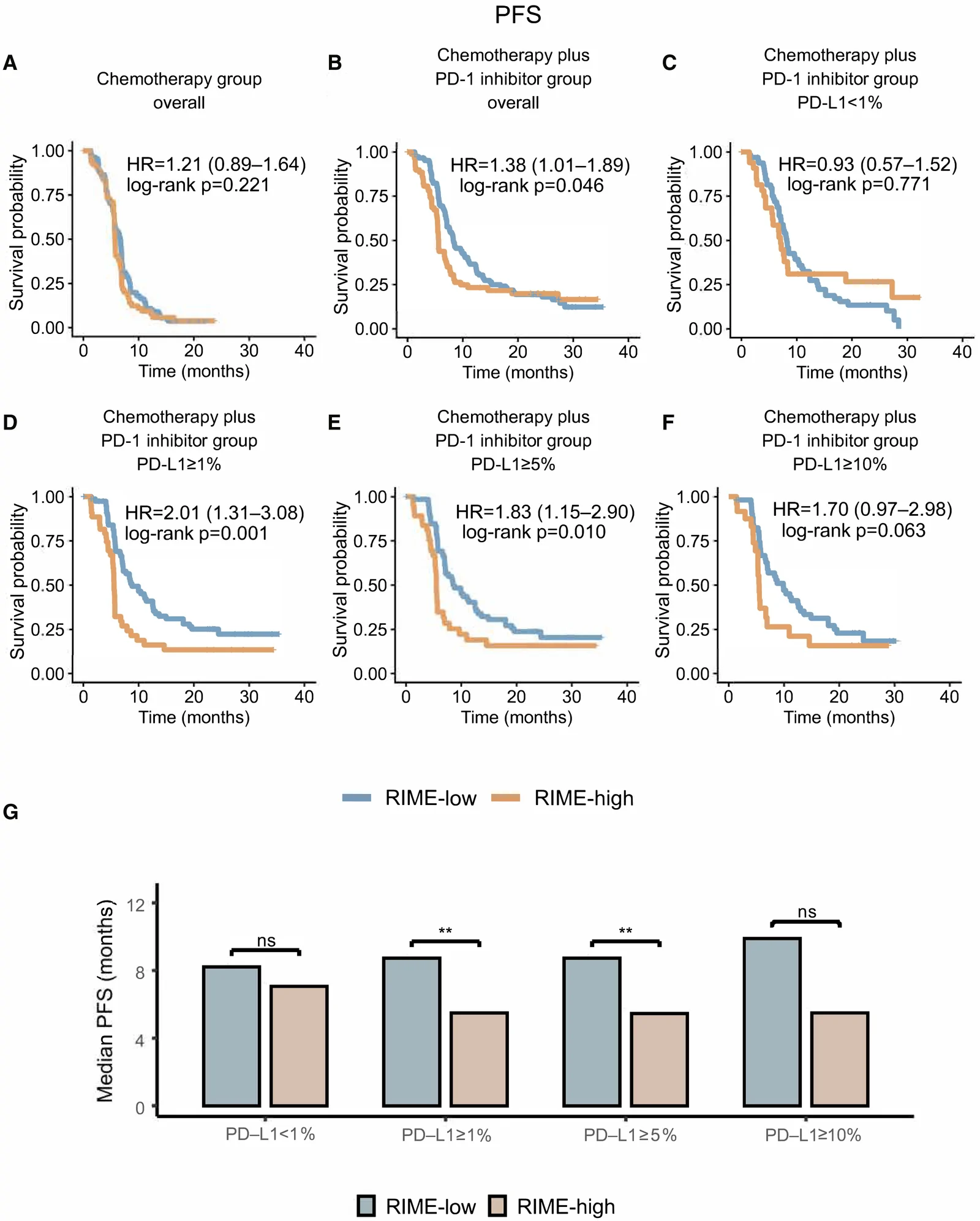
Progression-free survival by baseline RIME status across treatment groups and PD-L1-defined subgroups Kaplan-Meier curves show PFS according to RIME-low versus RIME-high status; *n* represents individual patients. HRs compare RIME-high with RIME-low and were estimated using univariable Cox proportional hazards models; 95% confidence intervals are shown in parentheses. *p* values were calculated using two-sided log rank tests. Tick marks indicate censored observations. (A) Chemotherapy group overall (*n* = 218): HR = 1.21 (95% CI, 0.89–1.64), *p* = 0.221. (B) Chemotherapy plus PD-1 inhibitor group overall (*n* = 229): HR = 1.38 (95% CI, 1.01–1.89), *p* = 0.046. (C) PD-L1 <1% subgroup (*n* = 101): HR = 0.93 (95% CI, 0.57–1.52), *p* = 0.771. (D) PD-L1 ≥1% subgroup (*n* = 124): HR = 2.01 (95% CI, 1.31–3.08), *p* = 0.001. (E) PD-L1 ≥5% subgroup (*n* = 107): HR = 1.83 (95% CI, 1.15–2.90), *p* = 0.010. (F) PD-L1 ≥10% subgroup (*n* = 77): HR = 1.70 (95% CI, 0.97–2.98), *p* = 0.063. (G) Median PFS by RIME status across PD-L1-defined subgroups. ns, *p* ≥ 0.05; ∗*p* < 0.05; ∗∗*p* < 0.01.

### Combined assessment of baseline RIME and PD-L1 in the chemotherapy plus PD-1 inhibitor group

To further evaluate the prognostic discrimination performance of joint assessment of plasma lncRNA RIME and PD-L1 in the chemotherapy plus PD-1 inhibitor group, we compared the PFS and OS risks of RIME-high versus RIME-low within different PD-L1-defined strata. Forest plot analysis showed that in the PD-L1 <1% stratum, RIME had limited ability to distinguish outcomes, with PFS and OS HRs of 0.93 and 1.02, respectively, neither reaching statistical significance (Figure 5A). In contrast, in the PD-L1 ≥1% and ≥5% strata, RIME-high was associated with worse PFS and OS: in the PD-L1 ≥1% stratum, the HRs for PFS and OS were 2.01 and 1.74, respectively; in the PD-L1 ≥5% stratum, the corresponding values were 1.83 and 1.73. In the PD-L1 ≥10% stratum, the OS discrimination effect was further strengthened (HR = 2.01, *p* = 0.012), while PFS showed a consistent directional trend (HR = 1.70, *p* = 0.063) (Figure 5A).

**Figure 5:**
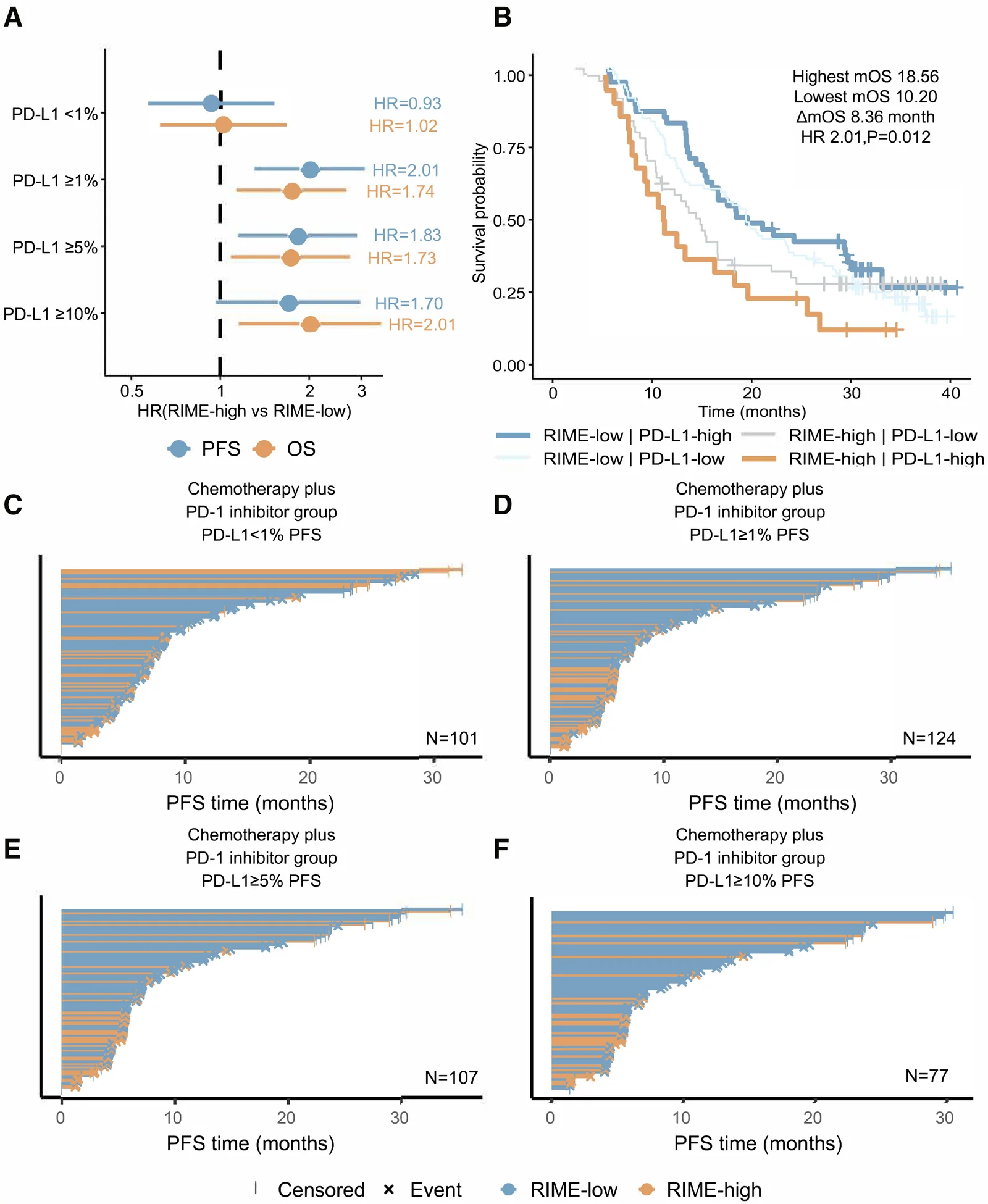
Combined assessment of baseline RIME and PD-L1 in the chemotherapy plus PD-1 inhibitor group (A) Forest plot of HRs for PFS and OS comparing RIME-high with RIME-low within PD-L1-defined subgroups. Blue indicates PFS and orange indicates OS; points indicate HRs and horizontal lines indicate 95% confidence intervals estimated from univariable Cox proportional hazards models. PFS/OS HRs were 0.93/1.02 for PD-L1 <1%, 2.01/1.74 for PD-L1 ≥1%, 1.83/1.73 for PD-L1 ≥5%, and 1.70/2.01 for PD-L1 ≥10%, respectively. (B) Kaplan-Meier estimates of OS across the four combined RIME/PD-L1 groups. PD-L1-high was defined as ≥10% and PD-L1-low as <10%. The HR and *p* value shown in the figure correspond to the comparison indicated in the figure; tick marks indicate censored observations. (C–F) Individual PFS event-time plots in the PD-L1 <1% (*n* = 101), ≥1% (*n* = 124), ≥5% (*n* = 107), and ≥10% (*n* = 77) subgroups, respectively. Each horizontal line represents one patient; vertical ticks indicate censoring and crosses indicate progression or death events. Blue denotes RIME-low and orange denotes RIME-high. *n* represents individual patients.

Consistently, OS Kaplan-Meier curves of the combined groups showed clearer prognostic separation across different RIME/PD-L1 combinations (Figure 5B). The RIME-low/PD-L1-high group had the numerically most favorable prognosis, with a median OS of 18.56 months, whereas the RIME-high/PD-L1-high group had the numerically poorest prognosis, with a median OS of 10.20 months; the difference between the two groups was 8.36 months (HR = 2.01, *p* = 0.012). Patient-level PFS trajectory plots further showed that longer progression-free durations were more commonly observed in the RIME-low group within the PD-L1 ≥1%, ≥5%, and ≥10% strata, whereas such differences were not evident in the PD-L1 <1% stratum (Figures 5C–5F).

Because multiple PD-L1 thresholds and clinical endpoints were examined, we performed an exploratory BH-FDR adjustment for 12 RIME-high versus RIME-low comparisons within the chemotherapy plus PD-1 inhibitor group, defined as four PD-L1 threshold-defined strata (<1%, ≥1%, ≥5%, and ≥10%) across ORR, PFS, and OS. All nominally significant subgroup associations remained statistically significant after adjustment (q < 0.05; Table S2).

Overall, these exploratory results suggest that the combination of plasma lncRNA RIME and PD-L1 may improve prognostic discrimination in the chemotherapy plus PD-1 inhibitor group and descriptively identify groups with more favorable or poorer outcomes.

### Independent outcome information of RIME beyond PD-L1

Baseline RIME itself stratified clinical outcomes in the chemotherapy plus PD-1 inhibitor group: compared with the RIME-high group, the RIME-low group showed a higher ORR and more favorable PFS and OS (Figures 2B, 3B, and 4B), supporting RIME as a prognostic outcome-stratification biomarker. To determine whether RIME provides outcome-discrimination information beyond PD-L1, rather than merely reclassifying PD-L1-defined groups, we performed nested model comparisons in the chemotherapy plus PD-1 inhibitor group. In the PD-L1 ≥1% subgroup, we first fitted a model containing the residual PD-L1 categories (1%–<5%, 5%–<10%, and ≥10%) and then assessed whether adding baseline RIME status improved model performance. Adding RIME improved discrimination for ORR (AUC, 0.611 to 0.773; likelihood-ratio test *p* = 2.24 × 10^−5^), PFS (Harrell’s C index, 0.523 to 0.604; *p* = 0.002), and OS (0.511–0.583; *p* = 0.013). Similar results were observed in the PD-L1 ≥5% subgroup (Figure S4). Corresponding analyses were not performed in the PD-L1 <1% or ≥10% subgroups because the available categorical PD-L1 variable had no residual variation within either subgroup; therefore, a PD-L1-only baseline model could not be constructed for comparison. These exploratory internal analyses indicate that RIME provides non-redundant outcome-discrimination information beyond PD-L1.

### Sensitivity analyses

To assess the robustness of the main findings and the exploratory RIME cutoff, we performed sensitivity analyses using multiple modeling approaches, including binary classification based on the analysis-derived cutoff, binary classification based on the median cutoff, tertile-based grouping, and analysis with plasma lncRNA RIME entered into the model as a continuous variable (Table S1). The direction of association was generally consistent across these approaches, although statistical strength was attenuated when alternative grouping strategies were used. We also performed exploratory bootstrap resampling of the cutoff-selection procedure to assess cutoff stability. The selected cutoff varied across bootstrap samples, indicating that the cutoff of 2.23 should be interpreted as an exploratory stratification threshold rather than a validated clinical decision threshold. These sensitivity analyses support the clinical outcome-stratification potential of plasma lncRNA RIME while highlighting the need for external validation of the cutoff in independent cohorts.

### Dynamic plasma lncRNA RIME changes

We further performed exploratory analyses of dynamic changes in plasma lncRNA RIME during treatment, evaluating the associations between post-treatment changes relative to baseline and treatment response and survival outcomes (Figures S2 and S3). Overall, the dynamic analyses showed a consistent direction, with the RIME-decline group tending to have better efficacy and prognosis than the RIME-increase group. Because some patients lacked complete longitudinal samples during treatment, the number of cases available for these analyses was limited, which may have reduced the statistical power; accordingly, these differences did not reach statistical significance. Nevertheless, these findings still suggest that dynamic changes in plasma lncRNA RIME may have potential clinical discriminatory value in ESCC.

### Treatment-by-RIME interaction analyses

To assess whether baseline RIME has predictive value for immunochemotherapy benefit, we fitted treatment-by-RIME interaction models. In the overall cohort and each PD-L1-defined subgroup, treatment effects of PD-1 inhibitor plus chemotherapy versus chemotherapy alone were estimated separately for RIME-low and RIME-high patients from the same model. The *p* value for the treatment-by-RIME interaction was used to determine whether these treatment effects differed between the two RIME groups. The treatment-by-RIME interaction for ORR was statistically significant in the overall cohort and in the PD-L1 ≥1%, ≥5%, and ≥10% subgroups. PFS interactions were also statistically significant in the PD-L1 ≥1%, ≥5%, and ≥10% subgroups (Figure S5). OS interaction estimates showed a consistent direction in the PD-L1 ≥1% (*p* = 0.216), ≥5% (*p* = 0.279), and ≥10% (*p* = 0.099) subgroups. These findings indicate that baseline RIME status provides an exploratory predictive signal for immunochemotherapy benefit.

### Multivariable analyses

To adjust for the potential effects of other clinical factors on outcomes, we fitted multivariable Cox models for PFS and OS and logistic regression models for ORR, adjusted for age, sex, ECOG performance status, disease stage, lymph-node status, metastatic burden, prior radiotherapy, and PD-L1 status. In the chemotherapy group, the multivariable results were consistent with the univariable analyses. In the chemotherapy plus PD-1 inhibitor group overall, RIME-high was significantly associated with lower ORR and inferior PFS and OS. These associations were also statistically significant in the PD-L1 ≥1%, ≥5%, and ≥10% subgroups (Table S3).

### Safety outcomes by RIME status

In the chemotherapy plus PD-1 inhibitor group, treatment-emergent adverse events (TEAEs) stratified by plasma lncRNA RIME, including common TEAEs occurring in at least 10% of patients in either group, are summarized in Table S4. Any-grade TEAEs occurred in 99.3% of patients in the RIME-low group and 100% of patients in the RIME-high group. The incidence of grade ≥3 TEAEs was similar between the RIME-low and RIME-high groups (75.7% vs 74.1%). Serious adverse events occurred in 39.9% and 37.0% of patients, respectively, and immune-related adverse events occurred in 87.8% and 82.7%, respectively. Treatment discontinuation due to adverse events was slightly more frequent in the RIME-high group than in the RIME-low group (11.1% vs 8.1%).

## Discussion

This exploratory biomarker analysis of a multicenter cohort yielded four main findings.First, PD-L1 alone had limited ability to distinguish clinical benefit with respect to efficacy and survival outcomes. Second, classification based on plasma lncRNA RIME expression distinguished groups with markedly different clinical benefit: in the chemotherapy plus PD-1 inhibitor group, RIME-low was associated with a higher ORR and better PFS/OS, whereas RIME-high was associated with poorer outcomes; when jointly evaluated with PD-L1, plasma lncRNA RIME more precisely distinguished patients with marked differences in clinical benefit, with the RIME-low/PD-L1-high and RIME-high/PD-L1-high groups showing the most favorable and poorest outcomes, respectively. Third, adverse events were generally comparable between the RIME-low and RIME-high groups, indicating that the observed associations of plasma lncRNA RIME were more apparent for efficacy and survival outcomes than for safety outcomes. Fourth, nested-model analyses indicated that RIME provides non-redundant outcome information beyond PD-L1 rather than merely repartitioning PD-L1-defined groups, while treatment-by-RIME interaction analyses provided an exploratory predictive signal for RIME, with the most consistent evidence observed for ORR and PFS.

Multiple phase III studies have established the overall benefit of PD-1 inhibitor plus chemotherapy in first-line treatment of ESCC.^4^^,^^5^^,^^6^^,^^9^^,^^14^ At the same time, heterogeneity in response remains evident at both the subgroup and individual-patient levels, with some patients achieving durable responses and others still experiencing early progression or limited benefit.^3^^,^^4^ In the present biomarker analysis, PD-L1 alone showed limited outcome discrimination within the immunochemotherapy-treated cohort under the evaluated threshold definitions.^15^^,^^20^ In our study, when PD-L1 was used alone, differences in ORR, PFS, and OS across threshold-defined strata were generally limited, supporting the notion that its ability to distinguish clinical benefit remains inadequate.

These limitations of PD-L1 alone provide the rationale for evaluating complementary biomarkers related to tumor immune regulation. Our previous study demonstrated that RIME promotes immune escape in ESCC by increasing H3K4me3 enrichment at the PD-L1 and IDO-1 promoters, thereby upregulating PD-L1 and IDO-1 expression, reducing intratumoral T cell infiltration and activity, and contributing to immunotherapy resistance in ESCC.^19^ Consistent with the biological context provided by our previous study, plasma lncRNA RIME was associated with differences in clinical outcomes. In the overall chemotherapy plus PD-1 inhibitor group, RIME-low was associated with a higher ORR and better PFS/OS, whereas RIME-high was associated with poorer outcomes. When examined across different PD-L1 threshold-defined strata, this distinction appeared more consistent in the PD-L1 ≥1%, ≥5%, and ≥10% strata. Notably, this association was observed across ORR, PFS, and OS rather than being limited to a single endpoint. After exploratory BH-FDR adjustment across the PD-L1 threshold-defined subgroup comparisons, all nominally significant associations remained statistically significant. Importantly, the ability of RIME to refine outcome stratification was not explained solely by PD-L1 categorization: nested-model analyses showed that RIME retained additional outcome information after residual PD-L1 categories were considered. Treatment-by-RIME interaction analyses further suggested that the relative benefit of PD-1 inhibitor plus chemotherapy differed by baseline RIME status, with the most consistent exploratory evidence observed for ORR and PFS. Together, these findings support plasma lncRNA RIME as an outcome-stratification biomarker with an exploratory predictive signal in patients receiving immunochemotherapy.

From a clinical perspective, integrating plasma lncRNA RIME with PD-L1 may improve discrimination of clinical benefit in patients receiving first-line immunochemotherapy for ESCC. These findings require further validation in external cohorts and prospective studies.

### Limitations of the study

This study has several limitations. First, the biomarker analysis was exploratory and involved repeated comparisons across multiple endpoints and PD-L1 threshold-defined strata without a prespecified confirmatory testing hierarchy. Second, the dichotomous cutoff for plasma lncRNA RIME was derived from the same cohort used for subsequent outcome analyses, which may introduce overfitting. Although sensitivity analyses and exploratory bootstrap assessment were performed, cutoff stability remained limited and alternative modeling strategies showed attenuated statistical strength. Therefore, the cutoff of 2.23 should be interpreted as an exploratory stratification threshold requiring independent validation rather than a definitive clinical cutoff or an approach for precise individual-level prediction. Third, although samples were obtained at multiple participating centers, all analyses were conducted in a single biomarker-evaluable cohort. The findings were therefore not evaluated in an independent external validation cohort and require validation in independent prospective multicenter cohorts. Fourth, because CPS was unavailable in the analyzed dataset, this study could not directly compare TPS- and CPS-based RIME/PD-L1 stratification or determine whether the observed associations would be reproduced under a CPS framework. Fifth, safety analyses were based mainly on comparisons of adverse-event incidence between RIME-defined groups and were not adjusted for treatment exposure time. In addition, the occurrence and reporting of adverse events may be influenced by follow-up duration and reporting bias. Thus, the relationship between plasma lncRNA RIME and specific adverse events requires further evaluation using more rigorous statistical modeling and independent data. Sixth, although sex was included as a covariate in multivariable models, sex-specific associations were not formally evaluated in this exploratory biomarker analysis. Therefore, potential differences in RIME-associated outcomes by sex require further investigation.

## Resource availability

### Lead contact

Further information and requests for resources and data should be directed to and will be fulfilled by the lead contact, Jia Liu (liujia1@sysucc.org.cn).

### Materials availability

This study did not generate new unique reagents, cell lines, plasmids, or animal models.

### Data and code availability

• Data: The clinical and biomarker data analyzed in this study contain patient-level information. De-identified data may be requested from the lead contact. Access is subject to approval by the relevant institutional review board, trial sponsor, and data access committee, where applicable.
• Analysis code used to generate the main statistical results and figures is publicly available at GitHub (https://github.com/Ricky1237788/RIME-ESCC-analysis) and archived on Zenodo (https://doi.org/10.5281/zenodo.22859798).
• Any additional information required to reanalyze the data reported in this study is available from the lead contact upon request.

## Acknowledgments

This work was supported by the Noncommunicable Chronic Diseases-National Science and Technology Major Project (2024ZD0533300); the 10.13039/501100001809National Natural Science Foundation of China (92374114, 82273402, 82321003, 82173128, 82203480); the 10.13039/501100003453Natural Science Foundation of Guangdong Province (2024A1515012888, 2014A030312015, 2019A1515011109); the 10.13039/501100021171Guangdong Basic and Applied Basic Research Foundation (2026A1515010717); the Science and Technology Program of Guangzhou (202002030208, 202206080008); and the Young Talents Program of Sun Yat-sen University Cancer Center (YTP-SYSUCC-0084, YTP-SYSUCC-0078).

## Author contributions

J.L., Q.-N.W., and H.-Y.L. conceived and supervised the study. J.L., R.-Q.R., X.-M.Z., and Y.H. performed the data analysis and drafted the manuscript. Q.-H.Z., X.-J.L., and Y.-Z.C. contributed to data collection and experimental procedures. All authors reviewed and approved the final version of the manuscript and take responsibility for the integrity of the work.

## Declaration of interests

The authors declare no competing interests.

## Declaration of generative AI and AI-assisted technologies in the manuscript preparation process

During the preparation of this work, the authors used AI to assist with language editing. After using these tools, the authors reviewed and edited the content as needed and take full responsibility for the content of the publication.

## STAR★Methods

### Key resources table

**Table:** 

REAGENT or RESOURCE	SOURCE	IDENTIFIER
**Biological samples**		

Pretreatment plasma samples from patients with advanced/metastatic ESCC	Previously conducted multicenter cohort	Available upon request from the lead contact

**Chemicals, peptides, and recombinant proteins**		

NucleoZOL reagent	MACHEREY-NAGEL	Cat#740404.200
Exogenous polyA spike-in control	TAKARA	N/A

**Critical commercial assays**		

PrimeScript RT Master Mix (Perfect Real Time)	Takara	Cat#RR036A
GoTaq qPCR Master Mix	Promega	Cat#A6002
PD-L1 immunohistochemistry kit/6E8 antibody	Abcam/Shuwen Biotech central laboratory	Cat#SAB028

**Software and algorithms**		

R statistical software	R Foundation for Statistical Computing	R.4.4.2
Original analysis code	GitHub/Zenodo	https://doi.org/10.5281/zenodo.22859798; https://github.com/Ricky1237788/RIME-ESCC-analysis
R packages for survival/statistical analysis and plotting	CRAN/Bioconductor	survival, survminer, timeROC/pROC, ggplot2, etc.

**Other**		

qPCR instrument	Roche	LightCycler 480 II

### Experimental model and study participant details

#### Study design and patients

This exploratory biomarker analysis used pretreatment plasma specimens and clinical data from a multicenter cohort involving patients with advanced or metastatic ESCC. The present analysis included 447 patients with available baseline plasma lncRNA RIME (LINC02096) expression data, comprising 218 patients in the chemotherapy group and 229 patients in the chemotherapy plus PD-1 inhibitor group. Patients in the chemotherapy plus PD-1 inhibitor group received first-line camrelizumab combined with paclitaxel and cisplatin, whereas those in the chemotherapy group received the same chemotherapy regimen without active PD-1 inhibition.

Patients were categorized into RIME-low and RIME-high groups using the exploratory, analysis-derived cutoff of 2.23 based on baseline polyA-normalized log RIME expression. In the chemotherapy group, 139 patients were classified as RIME-low and 79 as RIME-high. In the chemotherapy plus PD-1 inhibitor group, 148 patients were classified as RIME-low and 81 as RIME-high.

The main analysis population retained patients with non-missing baseline polyA-normalized log RIME expression. Patients were not excluded globally because of missing PFS, OS, ORR, or PD-L1 variables; each analysis excluded only patients missing variables required for that specific analysis.

#### Ethics approval and consent

The present biomarker analysis was conducted in accordance with the Declaration of Helsinki and applicable ethical requirements. The relevant clinical procedures and use of patient specimens were approved by the institutional review boards or independent ethics committees of the participating sites. Written informed consent was obtained from all participants.

### Method details

#### Plasma sample collection and RNA extraction

##### Baseline plasma samples were collected before treatment initiation

Plasma lncRNA RIME (LINC02096) expression was measured by real-time quantitative PCR. For each sample, 500 μL plasma was used for total RNA extraction. An exogenous polyA spike-in control (1.8×10ˆ8 copies/sample) was added before lysis for subsequent normalization. Samples were lysed in 1250 μL NucleoZOL reagent, followed by the addition of 500 μL RNase-free water, vigorous shaking for 15 s, incubation at room temperature for 5 min, and centrifugation at 12,000 g for 15 min. The supernatant was collected, and an equal volume of isopropanol was added to precipitate total RNA. The RNA pellet was washed with 75% ethanol, air-dried, and resuspended in 10 μL RNase-free water. RNA concentration and purity were measured using a NanoDrop microvolume spectrophotometer (Thermo Fisher Scientific), and A260/280 and A260/230 were recorded. The extraction workflow was based on the total RNA isolation principle for liquid samples using NucleoZOL.

#### Reverse transcription and quantitative PCR

Extracted RNA was reverse-transcribed in a 20 μL reaction using PrimeScript™ RT Master Mix (Perfect Real Time) (Takara, Cat# RR036A). The reverse-transcription products were diluted two-fold and used as templates for quantitative PCR.

Quantitative PCR was performed in 20 μL reactions on a 384-well real-time PCR platform using GoTaq® qPCR Master Mix (Promega, Cat# A6002) to detect RIME and the exogenous polyA spike-in control. The cycling conditions were as follows: an initial hot-start polymerase activation step at 95°C for 2 min, followed by 50 cycles of denaturation at 95°C for 15 s and combined annealing/extension at 60°C for 1 min. Each sample was analyzed in triplicate, and Ct values from the three technical replicate wells were averaged for downstream analysis.

RIME expression was normalized to the polyA spike-in control using the 2ˆ−ΔCt method, where ΔCt was calculated as Ct_RIME minus Ct_polyA. The normalized values were subsequently log-transformed to generate the baseline polyA-normalized log RIME expression value.

The primer sequences for RIME (LINC02096) were as follows: forward, 5′-TTCTGAGGCCCACGATAAGC-3′; reverse, 5′-TGGAGATGTGAGTTGCTGCC-3′.

#### RIME normalization and cutoff determination

Ct values from three technical replicates were first averaged for RIME and the polyA spike-in control. Relative RIME expression was then calculated using the 2^−ΔCt method and subsequently natural-log-transformed to generate the baseline polyA-normalized log RIME expression value used for RIME grouping.

The exploratory, analysis-derived RIME cutoff was 2.23. Patients with baseline polyA-normalized log RIME expression ≤2.23 were classified as RIME-low, and patients with baseline polyA-normalized log RIME expression > 2.23 were classified as RIME-high. The cutoff was derived using maximally selected rank statistics based on PFS time and event status. Sensitivity analyses and bootstrap resampling of the cutoff-selection procedure were performed to assess robustness and cutoff stability. The cutoff should be interpreted as an exploratory stratification threshold rather than a validated clinical decision threshold.

#### PD-L1 immunohistochemistry and TPS subgroup definition

PD-L1 expression was assessed by immunohistochemistry at a central laboratory (Shuwen Biotech, Deqing, Zhejiang, China) using a PD-L1 immunohistochemistry kit (6E8 antibody, Abcam). PD-L1 expression was characterized by tumor proportion score (TPS), defined as the percentage of viable tumor cells showing partial or complete membrane staining of at least 1+ among all viable tumor cells.

Nested TPS threshold definitions of PD-L1 <1%, ≥1%, ≥5%, and ≥10% were used for subgroup analyses. The ≥1%, ≥5%, and ≥10% thresholds were treated as nested comparisons, with PD-L1 <1% as the reference group for each threshold comparison. Patients were not analyzed as mutually exclusive four-level PD-L1 categories for the primary threshold analyses.

TPS was used because PD-L1 was assessed with the 6E8 assay and recorded using a tumor-cell TPS framework; CPS data were unavailable, and samples were not re-scored for CPS for the present biomarker analysis.^4^^,^^7^ This TPS-based approach is consistent with ESCC immunotherapy studies using tumor-cell PD-L1/TPS categories, including CheckMate 648 and TPS-stratified analyses of JUPITER-06, although other pivotal trials such as KEYNOTE-590 used CPS.^3^^,^^5^^,^^9^^,^^12^^,^^13^ Because TPS and CPS capture partly different components of PD-L1 staining, they should not be considered interchangeable.

PD-L1 TPS scoring was independently evaluated by two pathologists who had no access to treatment information, plasma RIME status, or clinical outcomes. Any scoring discrepancies were resolved by joint review and consensus. A formal numerical interobserver concordance estimate was not available for this retrospective analysis.

#### Clinical endpoints

The efficacy endpoints included objective response rate (ORR), progression-free survival (PFS), and overall survival (OS). ORR was defined as the proportion of patients with complete response or partial response. Stable disease and progressive disease were classified as non-response for ORR comparisons. PFS was measured from the original treatment-allocation date to disease progression or death from any cause. OS was measured from the original treatment-allocation date to death from any cause. Safety endpoints included treatment-emergent adverse events (TEAEs) and related outcomes.

#### Dynamic plasma RIME analysis

Exploratory dynamic analyses evaluated plasma lncRNA RIME at baseline, end of treatment (EOT), and ongoing-treatment timepoints using the corresponding polyA-normalized log RIME expression values. Dynamic analyses included only patients with available samples at the corresponding timepoint and did not affect the main baseline analysis population (Figures S2 and S3).

### Quantification and statistical analysis

#### General statistical approach

Baseline characteristics were summarized descriptively according to RIME group. Analyses were performed by treatment group, and PD-L1 subgroup analyses primarily focused on the chemotherapy plus PD-1 inhibitor group because the objective was to evaluate RIME in the context of PD-1 inhibitor plus chemotherapy. All tests were two-sided unless otherwise specified. Statistical analyses and visualizations were performed using R software (version 4.4.2; R Foundation for Statistical Computing, Vienna, Austria). Statistical details for individual analyses, including sample size, statistical tests, effect estimates, confidence intervals, and *p* values, are reported in the corresponding figure legends, figures, results, or STAR Methods, as appropriate.

#### ORR analysis

For ORR comparisons, response-evaluable patients were defined as those with investigator-assessed best overall response of complete response, partial response, stable disease, or progressive disease. Complete response and partial response were classified as responders, whereas stable disease and progressive disease were classified as non-responders. Patients without a post-baseline tumor assessment (NA) or with a non-evaluable response (NE) were excluded from ORR analyses. Between-group differences were assessed using two-sided Fisher’s exact tests. ORs and 95% CIs were reported where applicable, with RIME-high versus RIME-low used as the unified direction in multiplicity-adjustment tables.

#### Survival analysis

PFS and OS were estimated using the Kaplan-Meier method. Between-group differences were evaluated using the log-rank test. Hazard ratios and 95% confidence intervals were estimated using Cox proportional hazards models. For RIME group comparisons, HRs were reported for RIME-high versus RIME-low, such that HR > 1 indicates worse survival in the RIME-high group.

#### Multivariable analyses

To adjust for potential effects of other clinical factors on outcomes, multivariable Cox proportional hazards models were fitted separately in the chemotherapy plus PD-1 inhibitor group and the chemotherapy group for PFS and OS. Models included baseline RIME group, age, sex, ECOG performance status, disease stage, lymph-node status, metastatic burden, prior radiotherapy, and PD-L1 status. Multivariable logistic regression models using the same covariates were fitted for ORR. For analyses within PD-L1-defined subgroups, PD-L1 status was not included because it was fixed by subgroup definition. RIME effects were estimated as RIME-high versus RIME-low, with adjusted HRs or ORs and 95% CIs reported (Table S3).

#### Independent outcome information beyond PD-L1 analysis

To assess whether baseline RIME provided non-redundant outcome information beyond PD-L1 in PD-L1-positive patients in the chemotherapy plus PD-1 inhibitor group, nested models were compared within PD-L1 ≥1% and PD-L1 ≥5% subgroups. The baseline models included residual PD-L1 categories within the relevant positive subgroup, and the full models additionally included RIME group. Analogous comparisons were not performed in the PD-L1 <1% or ≥10% subgroups because the available categorical PD-L1 variable had no residual variation within either subgroup. For ORR, nested logistic regression models were compared using likelihood-ratio tests and apparent area under the receiver-operating-characteristic curve (AUC). For PFS and OS, nested Cox proportional hazards models were compared using likelihood-ratio tests and apparent Harrell’s C-index. These internal performance estimates are exploratory and require external validation (Figure S4).

#### Treatment×RIME interaction analysis

To assess treatment-predictive potential, unadjusted treatment-by-RIME interaction models were fitted with treatment group, RIME group, and their interaction term. For PFS and OS, Cox proportional hazards models were used to estimate interaction HRs, 95% CIs, and Wald *p* values. For ORR, logistic regression models were used to estimate interaction ORs, 95% CIs, and Wald *p* values. Within each model, treatment effects of the chemotherapy plus PD-1 inhibitor group versus the chemotherapy group were estimated separately for RIME-low and RIME-high patients, and the interaction P value was used to assess whether these treatment effects differed between the two RIME groups (Figure S5).

#### Multiplicity adjustment

Given the exploratory nature of the biomarker analyses, no confirmatory multiplicity-testing hierarchy was applied. All P values are nominal unless otherwise specified. To address multiplicity within the PD-L1 subgroup analyses, the Benjamini-Hochberg false-discovery-rate (BH-FDR) method was applied to 12 RIME-high versus RIME-low comparisons within the chemotherapy plus PD-1 inhibitor group, defined as four PD-L1 threshold-defined strata (<1%, ≥1%, ≥5%, and ≥10%) across ORR, PFS, and OS. Overall comparisons within the chemotherapy plus PD-1 inhibitor group and the chemotherapy group were reported with nominal P values and were not included in the subgroup-focused BH-FDR adjustment. FDR-adjusted q values for all 12 subgroup comparisons within the chemotherapy plus PD-1 inhibitor group are provided in Table S2. These subgroup findings were interpreted as hypothesis-generating rather than confirmatory.

#### Cutoff sensitivity analyses

Sensitivity analyses were performed using multiple modeling approaches, including binary classification based on the analysis-derived cutoff, binary classification based on the median cutoff, tertile-based grouping, and analysis with plasma lncRNA RIME entered as a continuous variable. Exploratory bootstrap resampling of the cutoff-selection procedure was used to assess cutoff stability, with results reported in Table S1.

### Additional resources

Requests for access to the data supporting this study should be directed to the lead contact, subject to applicable institutional and ethical approvals.
