# Supplementary material for: A biomarker analysis of plasma RIME and PD-L1 for stratifying first-line immunochemotherapy outcomes in advanced ESCC
Source: iScience. 2026 Oct 23;29(11):117995. doi: 10.1016/j.isci.2026.117995 (PMC13720606; doi:10.1016/j.isci.2026.117995)
Supplement: Supplementary file 1 [file mmc1.pdf]

iScience, Volume *29*

Supplemental information

A biomarker analysis of plasma RIME and PD-L1 for stratifying first-line immunochemotherapy outcomes in advanced ESCC

Jia Liu, Rui-Qi Ren, Ye He, Qi-Hua Zhang, Xiao-Min Zhang, Xiao-Jing Luo, Yu-Zhe Cao, Qi-Nian Wu, and Hui-Yan Luo

Figure S1 Time-dependent ROC anchored at 6-month PFS (PFS6m) related to STAR Methods

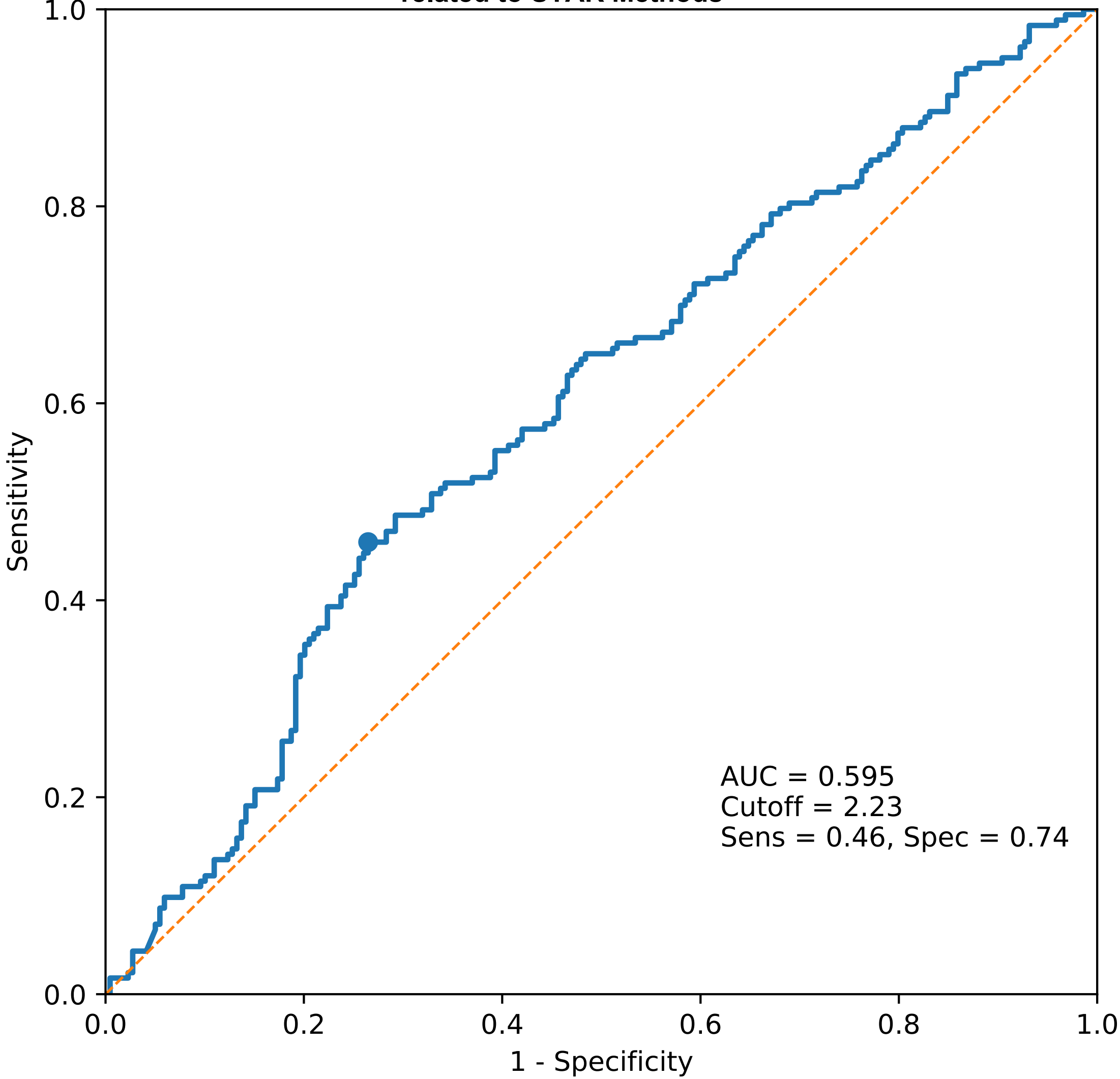

Figure S2 Dynamic plasma lncRNA RIME: ONGOING-Baseline / Baseline (related to STAR Methods)

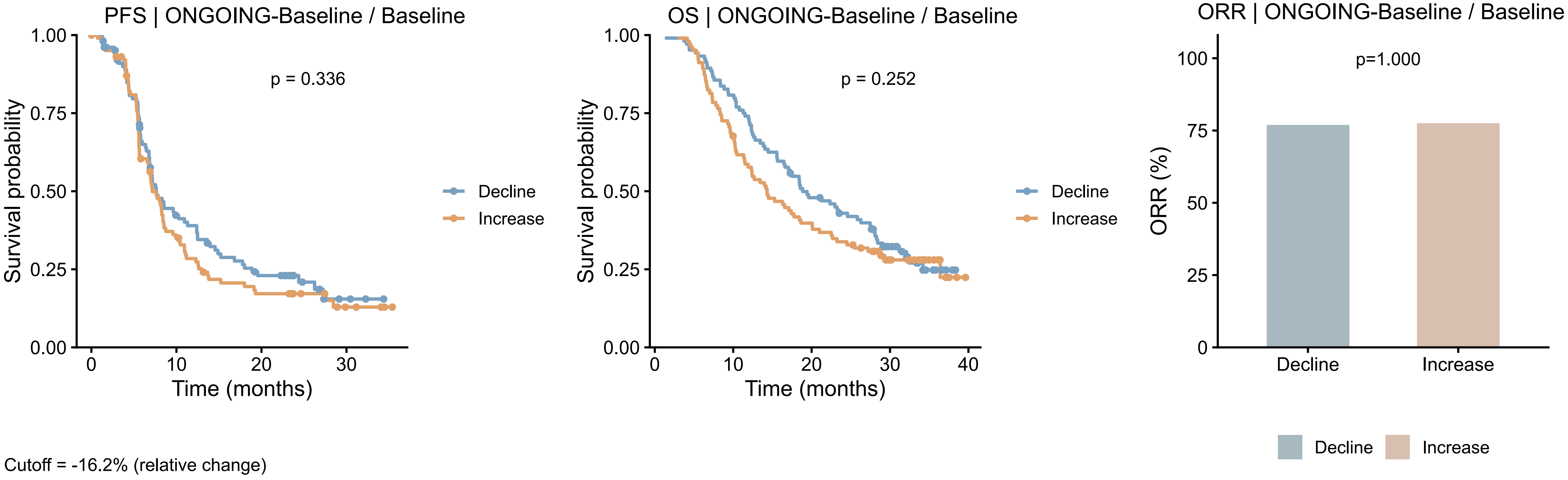

Cutoff = -16.2% (relative change)

Figure S3 Joint analysis of PD-L1 strata and dynamic plasma lncRNA RIME (related to STAR Methods)

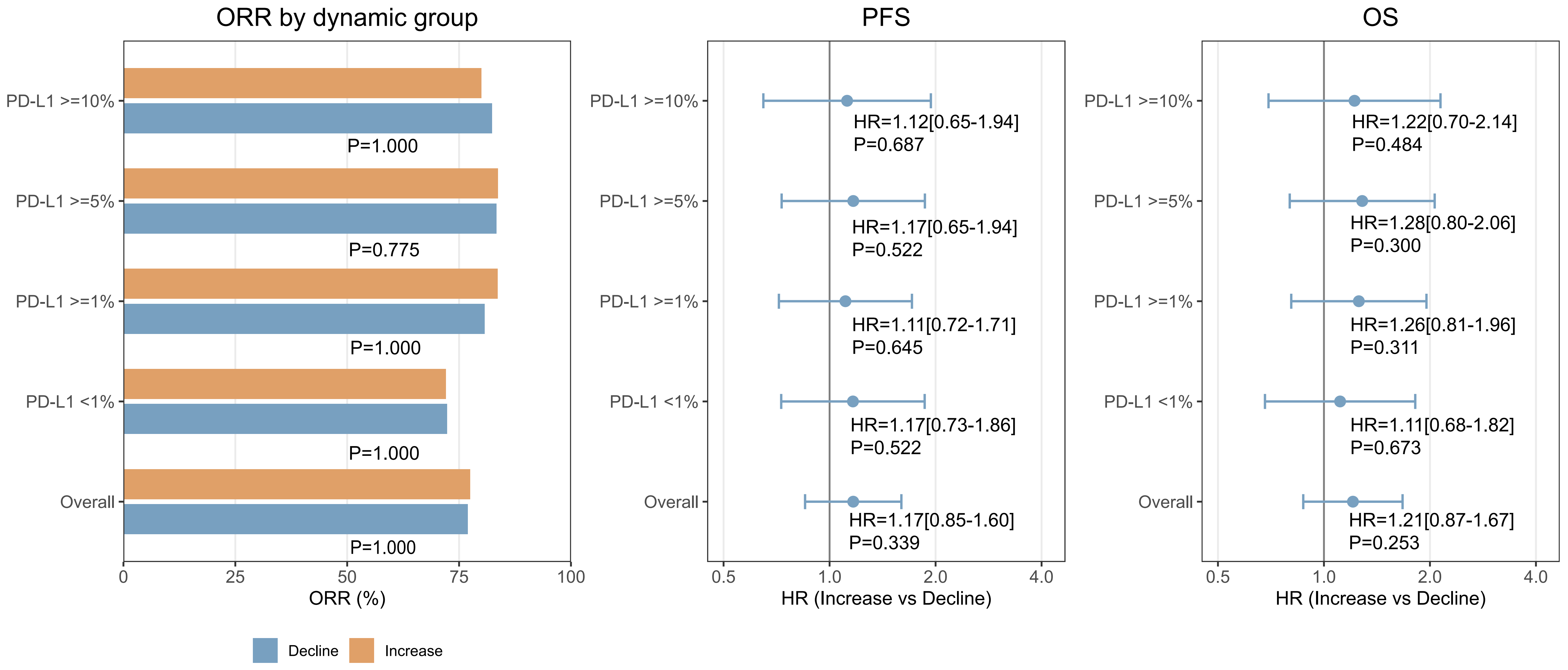

Formula: (ONGOING-Baseline)/Baseline; Decline <= cutoff, Increase > cutoff.

Figure S4 Incremental outcome discrimination provided by RIME beyond residual PD-L1 information related to Figure 5 and STAR Methods

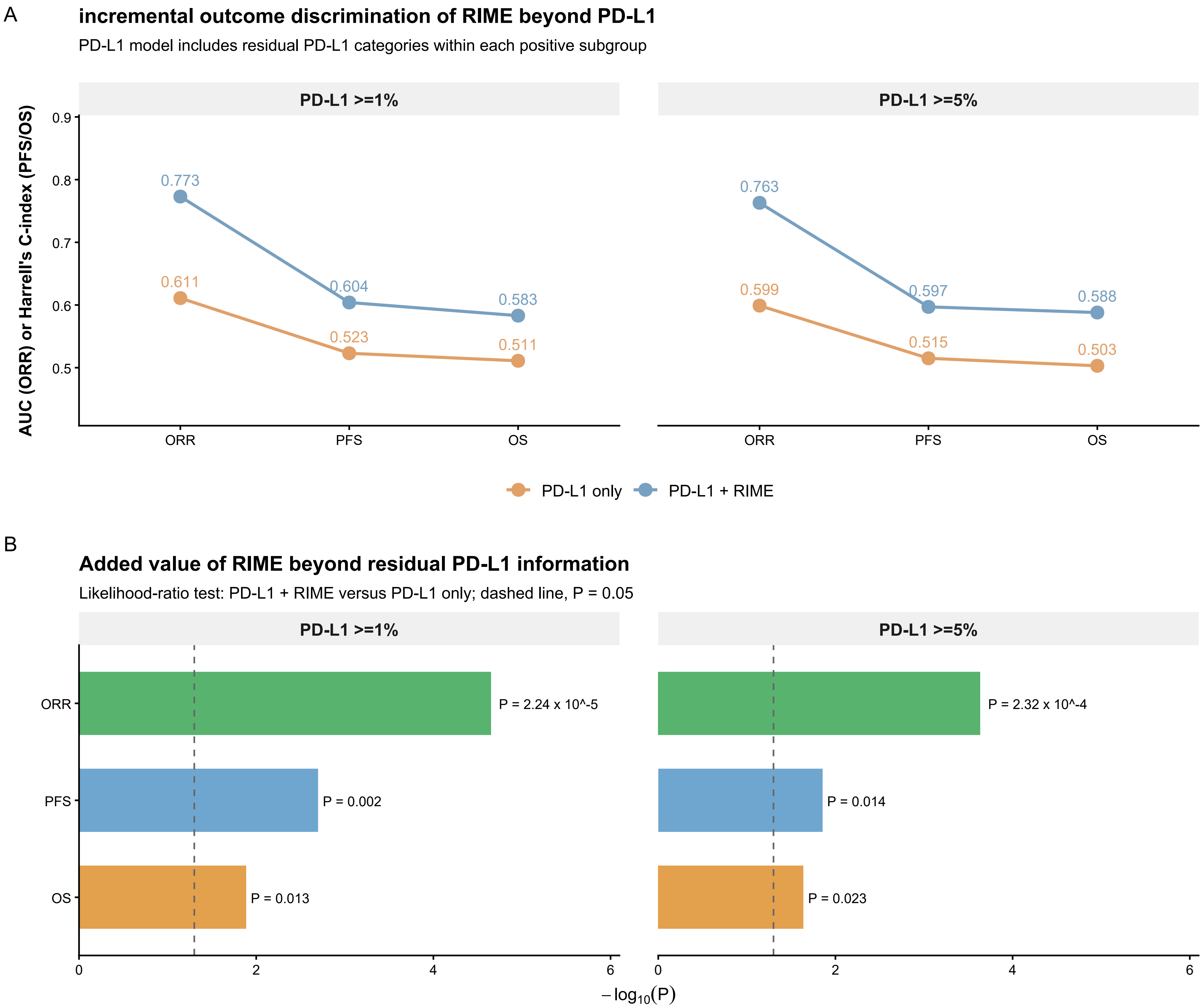

Figure S5 Treatment effects of PD−1 inhibitor plus chemotherapy by RIME status related to Figures 2–4 and STAR Methods

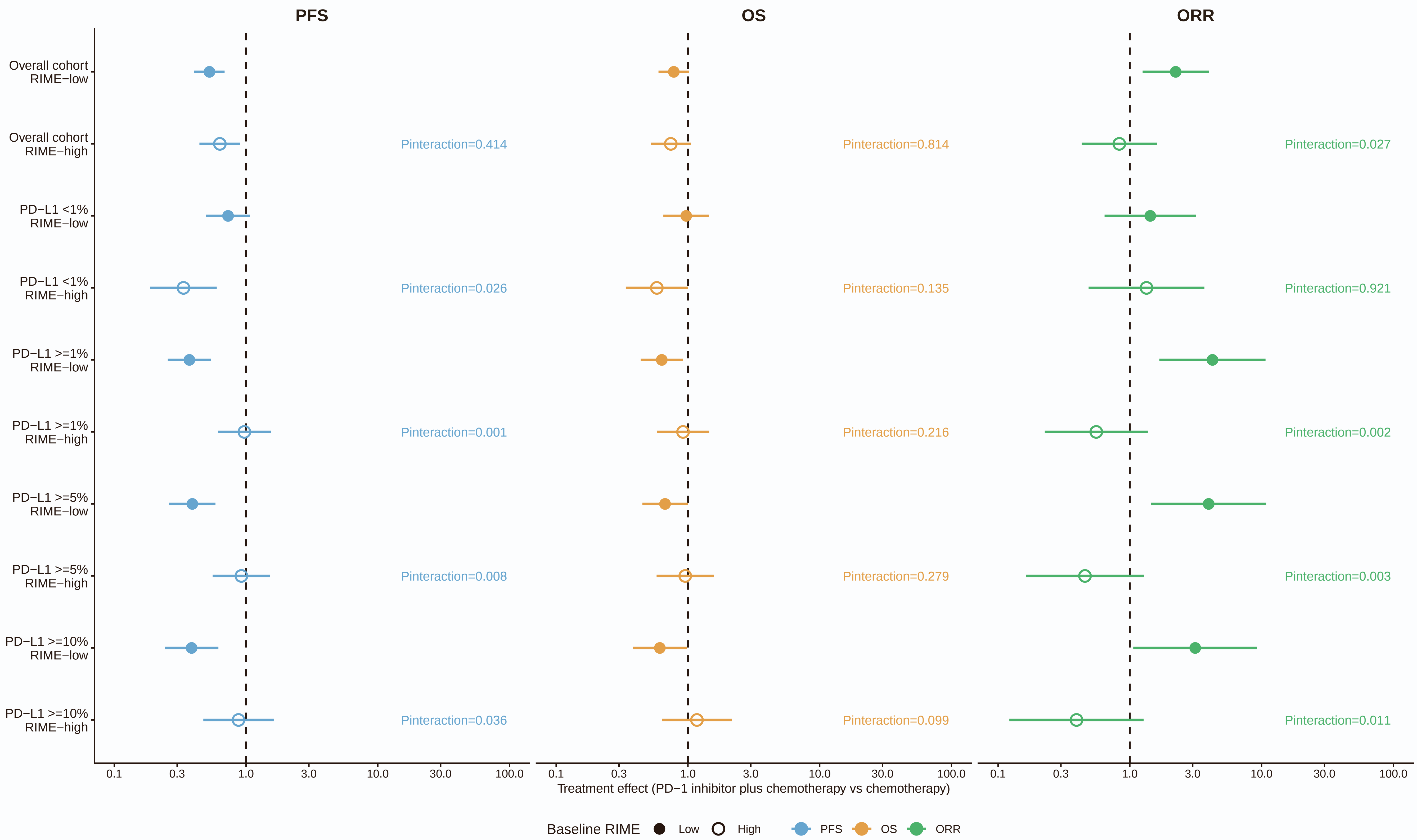

Table S1. Sensitivity analyses for RIME parameterization (related to STAR Methods)

Progression-free survival (PFS)

Subgroup	Model	HR (95% CI)	P value	N	Events
Chemotherapy group overall	Binary (Median cutoff)	1.01 (0.75-1.35)	0.944	218	181
Chemotherapy group overall	Binary (analysis-derived cutoff,	1.21 (0.89-1.64)	0.226	218	181
Chemotherapy group overall	Continuous (per SD increase)	0.98 (0.85-1.12)	0.733	218	181
Chemotherapy group overall	Tertile trend (1-3)	1.09 (0.91-1.30)	0.336	218	181
Chemotherapy plus PD-1 inhibitor group, PD-L1 <1%	Binary (Median cutoff)	1.01 (0.64-1.57)	0.980	101	79
Chemotherapy plus PD-1 inhibitor group, PD-L1 <1%	Binary (analysis-derived cutoff,	0.93 (0.57-1.52)	0.772	101	79
Chemotherapy plus PD-1 inhibitor group, PD-L1 <1%	Continuous (per SD increase)	0.95 (0.75-1.20)	0.650	101	79
Chemotherapy plus PD-1 inhibitor group, PD-L1 <1%	Tertile trend (1-3)	0.95 (0.72-1.26)	0.722	101	79
Chemotherapy plus PD-1 inhibitor group, PD-L1 >=1%	Binary (Median cutoff)	1.31 (0.87-1.98)	0.202	124	90
Chemotherapy plus PD-1 inhibitor group, PD-L1 >=1%	Binary (analysis-derived cutoff,	1.99 (1.30-3.06)	0.002	124	90
Chemotherapy plus PD-1 inhibitor group, PD-L1 >=1%	Continuous (per SD increase)	1.33 (1.05-1.67)	0.017	124	90
Chemotherapy plus PD-1 inhibitor group, PD-L1 >=1%	Tertile trend (1-3)	1.41 (1.06-1.86)	0.017	124	90
Chemotherapy plus PD-1 inhibitor group, PD-L1 >=10%	Binary (Median cutoff)	0.99 (0.59-1.67)	0.983	77	58
Chemotherapy plus PD-1 inhibitor group, PD-L1 >=10%	Binary (analysis-derived cutoff,	1.69 (0.96-2.97)	0.067	77	58
Chemotherapy plus PD-1 inhibitor group, PD-L1 >=10%	Continuous (per SD increase)	1.14 (0.83-1.58)	0.408	77	58
Chemotherapy plus PD-1 inhibitor group, PD-L1 >=10%	Tertile trend (1-3)	1.27 (0.88-1.82)	0.203	77	58
Chemotherapy plus PD-1 inhibitor group, PD-L1 >=5%	Binary (Median cutoff)	1.16 (0.74-1.81)	0.519	107	78
Chemotherapy plus PD-1 inhibitor group, PD-L1 >=5%	Binary (analysis-derived cutoff,	1.82 (1.14-2.89)	0.011	107	78
Chemotherapy plus PD-1 inhibitor group, PD-L1 >=5%	Continuous (per SD increase)	1.26 (0.98-1.62)	0.072	107	78
Chemotherapy plus PD-1 inhibitor group, PD-L1 >=5%	Tertile trend (1-3)	1.32 (0.97-1.78)	0.076	107	78
Chemotherapy plus PD-1 inhibitor group overall	Binary (Median cutoff)	1.18 (0.87-1.59)	0.277	229	172
Chemotherapy plus PD-1 inhibitor group overall	Binary (analysis-derived cutoff,	1.38 (1.00-1.89)	0.048	229	172
Chemotherapy plus PD-1 inhibitor group overall	Continuous (per SD increase)	1.10 (0.94-1.30)	0.240	229	172
Chemotherapy plus PD-1 inhibitor group overall	Tertile trend (1-3)	1.16 (0.95-1.41)	0.144	229	172

Hazard ratios (HRs) were estimated using univariable Cox proportional hazards models within each subgroup. Binary models compare RIME-high versus RIME-low; continuous models are per SD increase in logpoly_RNA_ave; tertile trend treats tertile as an ordinal variable (1-3).

Overall survival (OS)

Subgroup	Model	HR (95% CI)	P value	N	Events
Chemotherapy group overall	Binary (Median cutoff)	1.35 (1.01-1.82)	0.045	218	179
Chemotherapy group overall	Binary (analysis-derived cutoff,	1.42 (1.05-1.91)	0.024	218	179
Chemotherapy group overall	Continuous (per SD increase)	1.13 (0.99-1.29)	0.061	218	179
Chemotherapy group overall	Tertile trend (1-3)	1.28 (1.07-1.52)	0.007	218	179
Chemotherapy plus PD-1 inhibitor group, PD-L1 <1%	Binary (Median cutoff)	0.94 (0.60-1.48)	0.789	101	74
Chemotherapy plus PD-1 inhibitor group, PD-L1 <1%	Binary (analysis-derived cutoff,	1.02 (0.62-1.67)	0.939	101	74
Chemotherapy plus PD-1 inhibitor group, PD-L1 <1%	Continuous (per SD increase)	0.94 (0.74-1.19)	0.613	101	74
Chemotherapy plus PD-1 inhibitor group, PD-L1 <1%	Tertile trend (1-3)	0.97 (0.72-1.29)	0.815	101	74
Chemotherapy plus PD-1 inhibitor group, PD-L1 >=1%	Binary (Median cutoff)	1.44 (0.95-2.19)	0.086	124	89
Chemotherapy plus PD-1 inhibitor group, PD-L1 >=1%	Binary (analysis-derived cutoff,	1.74 (1.13-2.67)	0.011	124	89
Chemotherapy plus PD-1 inhibitor group, PD-L1 >=1%	Continuous (per SD increase)	1.30 (1.03-1.65)	0.025	124	89
Chemotherapy plus PD-1 inhibitor group, PD-L1 >=1%	Tertile trend (1-3)	1.29 (0.98-1.68)	0.067	124	89
Chemotherapy plus PD-1 inhibitor group, PD-L1 >=10%	Binary (Median cutoff)	1.45 (0.85-2.46)	0.173	77	55
Chemotherapy plus PD-1 inhibitor group, PD-L1 >=10%	Binary (analysis-derived cutoff,	2.01 (1.15-3.50)	0.014	77	55
Chemotherapy plus PD-1 inhibitor group, PD-L1 >=10%	Continuous (per SD increase)	1.30 (0.96-1.77)	0.093	77	55
Chemotherapy plus PD-1 inhibitor group, PD-L1 >=10%	Tertile trend (1-3)	1.37 (0.96-1.95)	0.085	77	55
Chemotherapy plus PD-1 inhibitor group, PD-L1 >=5%	Binary (Median cutoff)	1.40 (0.90-2.20)	0.139	107	77
Chemotherapy plus PD-1 inhibitor group, PD-L1 >=5%	Binary (analysis-derived cutoff,	1.73 (1.08-2.75)	0.021	107	77
Chemotherapy plus PD-1 inhibitor group, PD-L1 >=5%	Continuous (per SD increase)	1.29 (1.01-1.65)	0.043	107	77
Chemotherapy plus PD-1 inhibitor group, PD-L1 >=5%	Tertile trend (1-3)	1.29 (0.96-1.73)	0.086	107	77
Chemotherapy plus PD-1 inhibitor group overall	Binary (Median cutoff)	1.20 (0.89-1.63)	0.240	229	166
Chemotherapy plus PD-1 inhibitor group overall	Binary (analysis-derived cutoff,	1.32 (0.96-1.82)	0.084	229	166
Chemotherapy plus PD-1 inhibitor group overall	Continuous (per SD increase)	1.09 (0.93-1.28)	0.288	229	166
Chemotherapy plus PD-1 inhibitor group overall	Tertile trend (1-3)	1.12 (0.92-1.36)	0.260	229	166

Same modeling strategy as above. P values are nominal (two-sided).

Table S2. Exploratory BH-FDR adjustment for RIME-high versus RIME-low comparisons across PD-L1-defined subgroups (related to Figures 2–4 and STAR Methods)

endpoint	pd_l1_stratum	compariso	n	events_or_	effect_measure	effect_estimate	effect_95CI	display_effect	nominal_p
ORR	PD-L1 <1%	RIME-high vs RIME-low	101	71	OR for response (RIME-high vs RIME-low)	0.528642	0.192042-1.463438	OR=0.53 (0.19-1.46); ORR 21/33 vs 50/65	0.231
PFS	PD-L1 <1%	RIME-high vs RIME-low	101	79 events	HR (RIME-high vs RIME-low)	0.93	0.57-1.52	HR=0.93 (0.57-1.52)	0.770
OS	PD-L1 <1%	RIME-high vs RIME-low	101	74 events	HR (RIME-high vs RIME-low)	1.02	0.62-1.67	HR=1.02 (0.62-1.67)	0.938
ORR	PD-L1 >=1%	RIME-high vs RIME-low	124	98	OR for response (RIME-high vs RIME-low)	0.151452	0.047346-0.436504	OR=0.15 (0.05-0.44); ORR 26/43 vs 72/79	<0.001
PFS	PD-L1 >=1%	RIME-high vs RIME-low	124	90 events	HR (RIME-high vs RIME-low)	2.007077	1.31-3.08	HR=2.01 (1.31-3.08)	0.001
OS	PD-L1 >=1%	RIME-high vs RIME-low	124	89 events	HR (RIME-high vs RIME-low)	1.7426368	1.13-2.68	HR=1.74 (1.13-2.68)	0.011
ORR	PD-L1 >=5%	RIME-high vs RIME-low	107	86	OR for response (RIME-high vs RIME-low)	0.171879	0.047583-0.552577	OR=0.17 (0.05-0.55); ORR 23/36 vs 63/69	0.001
PFS	PD-L1 >=5%	RIME-high vs RIME-low	107	78 events	HR (RIME-high vs RIME-low)	1.8262114	1.15-2.90	HR=1.83 (1.15-2.90)	0.011
OS	PD-L1 >=5%	RIME-high vs RIME-low	107	77 events	HR (RIME-high vs RIME-low)	1.727034	1.09-2.75	HR=1.73 (1.09-2.75)	0.021
ORR	PD-L1 >=10%	RIME-high vs RIME-low	77	60	OR for response (RIME-high vs RIME-low)	0.1508704	0.05-0.49	OR=0.15 (0.05-0.49); ORR 13/24 vs 47/53	0.002
PFS	PD-L1 >=10%	RIME-high vs RIME-low	77	58 events	HR (RIME-high vs RIME-low)	1.6991065	0.97-2.98	HR=1.70 (0.97-2.98)	0.065
OS	PD-L1 >=10%	RIME-high vs RIME-low	77	55 events	HR (RIME-high vs RIME-low)	2.0095799	1.15-3.50	HR=2.01 (1.15-3.50)	0.014

BH-FDR adjustment was applied to all 12 PD-L1 subgroup comparisons within the chemotherapy plus PD-1 inhibitor group: four PD-L1 threshold-defined strata (<1%, >=1%, >=5%, and >=10%) × three endpoints (ORR, PFS, and OS). For ORR, nominal P values were from two-sided Fisher’s exact tests; for PFS and OS, nominal P values were Wald tests from univariable Cox proportional hazards models. Overall comparisons in the chemotherapy plus PD-1 inhibitor and chemotherapy groups were not included in this adjustment.

Table S3. Multivariable associations of baseline RIME with ORR, PFS, and OS by treatment group and PD-L1 subgroup (related to Figures 2–4 and STAR Methods)

Complete-case analyses. Multivariable Cox proportional hazards models were used for PFS and OS, and multivariable logistic regression was used for ORR.

Treatment group	PD-L1 subgroup	Endpoint	n	Events/responders	Adjusted effect (95% CI)	P value	Model
Chemotherapy group	Overall	ORR	213	143 responders	OR=0.72 (0.39-1.33)	0.293	Logistic regression
Chemotherapy group	Overall	PFS	213	176 events	HR=1.25 (0.89-1.74)	0.195	Cox regression
Chemotherapy group	Overall	OS	213	175 events	HR=1.41 (1.03-1.93)	0.034	Cox regression
Chemotherapy plus PD-1 inhibitor group	Overall	ORR	225	169 responders	OR=0.22 (0.11-0.43)	<0.001	Logistic regression
Chemotherapy plus PD-1 inhibitor group	Overall	PFS	225	169 events	HR=1.54 (1.10-2.16)	0.012	Cox regression
Chemotherapy plus PD-1 inhibitor group	Overall	OS	225	163 events	HR=1.50 (1.07-2.09)	0.018	Cox regression
Chemotherapy plus PD-1 inhibitor group	PD-L1 <1%	ORR	98	71 responders	OR=0.33 (0.11-0.98)	0.045	Logistic regression
Chemotherapy plus PD-1 inhibitor group	PD-L1 <1%	PFS	101	79 events	HR=1.11 (0.64-1.92)	0.706	Cox regression
Chemotherapy plus PD-1 inhibitor group	PD-L1 <1%	OS	101	74 events	HR=1.16 (0.69-1.94)	0.583	Cox regression
Chemotherapy plus PD-1 inhibitor group	PD-L1 >=1%	ORR	122	98 responders	OR=0.11 (0.04-0.31)	<0.001	Logistic regression
Chemotherapy plus PD-1 inhibitor group	PD-L1 >=1%	PFS	124	90 events	HR=2.24 (1.42-3.54)	<0.001	Cox regression
Chemotherapy plus PD-1 inhibitor group	PD-L1 >=1%	OS	124	89 events	HR=2.03 (1.28-3.21)	0.003	Cox regression
Chemotherapy plus PD-1 inhibitor group	PD-L1 >=5%	ORR	105	86 responders	OR=0.10 (0.03-0.36)	<0.001	Logistic regression
Chemotherapy plus PD-1 inhibitor group	PD-L1 >=5%	PFS	107	78 events	HR=2.20 (1.34-3.60)	0.002	Cox regression
Chemotherapy plus PD-1 inhibitor group	PD-L1 >=5%	OS	107	77 events	HR=2.18 (1.32-3.60)	0.002	Cox regression
Chemotherapy plus PD-1 inhibitor group	PD-L1 >=10%	ORR	77	60 responders	OR=0.08 (0.02-0.35)	<0.001	Logistic regression
Chemotherapy plus PD-1 inhibitor group	PD-L1 >=10%	PFS	77	58 events	HR=2.09 (1.14-3.85)	0.018	Cox regression
Chemotherapy plus PD-1 inhibitor group	PD-L1 >=10%	OS	77	55 events	HR=2.28 (1.27-4.09)	0.006	Cox regression

All effects compare RIME-high with RIME-low. Overall treatment-group models were adjusted for age, sex, ECOG performance status, disease stage, lymph-node status, metastatic burden, prior radiotherapy, and PD-L1 status. PD-L1 status was not included in the PD-L1-defined subgroup models because it was fixed by subgroup definition. For ORR, responders were patients with complete or partial response; OR <1 indicates lower odds of response in the RIME-high group. For PFS and OS, HR >1 indicates poorer outcomes in the RIME-high group. n denotes the number of complete cases included in each model. PD-L1 >=1%, >=5%, and >=10% subgroups are nested.

Table S4. Treatment-emergent adverse events in the chemotherapy plus PD-1 inhibitor group by RIME group (related to STAR Methods)

Patients in the chemotherapy plus PD-1 inhibitor group with available RIME measurements. Values are n/N (%) for summary rows and n (%) for individual TEAEs. Grade ≥3 refers to CTCAE grade 3–5.

	**RIME Low (N=148)**		**RIME High (N=81)**	
**Adverse event**	**Any grade**	**Grade ≥3**	**Any grade**	**Grade ≥3**
**Summary**				
Any TEAE	147/148 (99.3)		81/81 (100.0)	
Grade ≥3 TEAE		112/148 (75.7)		60/81 (74.1)
Serious AE (SAE)	59/148 (39.9)		30/81 (37.0)	
Immune-related AE (irAE)	130/148 (87.8)		67/81 (82.7)	
AESI	19/148 (12.8)		8/81 (9.9)	
Steroid use (AEHORTH)	33/148 (22.3)		6/81 (7.4)	
AE leading to discontinuation (AEDIS)	12/148 (8.1)		9/81 (11.1)	
**Most common TEAEs (≥10% in either RIME group)**				
Anemia	126 (85.1)	35 (23.6)	68 (84.0)	19 (23.5)
Nausea	83 (56.1)	1 (0.7)	38 (46.9)	2 (2.5)
Neutrophil count decreased	84 (56.8)	51 (34.5)	41 (50.6)	26 (32.1)
White blood cell count decreased	64 (43.2)	21 (14.2)	42 (51.9)	13 (16.0)
Alopecia	70 (47.3)	1 (0.7)	35 (43.2)	0 (0.0)
Fatigue	68 (45.9)	4 (2.7)	33 (40.7)	0 (0.0)
Vomiting	66 (44.6)	4 (2.7)	41 (50.6)	5 (6.2)
Hypoalbuminemia	41 (27.7)	0 (0.0)	35 (43.2)	0 (0.0)
Weight decreased	49 (33.1)	2 (1.4)	27 (33.3)	1 (1.2)
Hyponatremia	42 (28.4)	21 (14.2)	22 (27.2)	11 (13.6)
Constipation	47 (31.8)	0 (0.0)	18 (22.2)	0 (0.0)
Hypokalemia	43 (29.1)	16 (10.8)	19 (23.5)	5 (6.2)
Platelet count decreased	31 (20.9)	4 (2.7)	20 (24.7)	3 (3.7)
Reactive cutaneous capillary endothelial proliferation	48 (32.4)	0 (0.0)	33 (40.7)	0 (0.0)
Reactive cutaneous capillary endothelial proliferation	80 (54.1)	1 (0.7)	30 (37.0)	1 (1.2)
Diarrhea	33 (22.3)	0 (0.0)	12 (14.8)	2 (2.5)
Pyrexia	28 (18.9)	0 (0.0)	11 (13.6)	0 (0.0)
White blood cell count decreased	34 (23.0)	10 (6.8)	12 (14.8)	2 (2.5)
Hyperuricemia	30 (20.3)	2 (1.4)	14 (17.3)	1 (1.2)
Fatigue	14 (9.5)	0 (0.0)	11 (13.6)	3 (3.7)
Hypoproteinemia	25 (16.9)	0 (0.0)	11 (13.6)	0 (0.0)
Decreased appetite	17 (11.5)	1 (0.7)	10 (12.3)	0 (0.0)
Hypochloremia	15 (10.1)	3 (2.0)	7 (8.6)	2 (2.5)
Hypocalcemia	18 (12.2)	1 (0.7)	9 (11.1)	2 (2.5)
Blood creatinine increased	25 (16.9)	1 (0.7)	11 (13.6)	0 (0.0)
Cough	34 (23.0)	0 (0.0)	8 (9.9)	0 (0.0)
Blood creatinine increased	14 (9.5)	0 (0.0)	10 (12.3)	1 (1.2)
Pneumonia	24 (16.2)	8 (5.4)	5 (6.2)	1 (1.2)
White blood cell count decreased	16 (10.8)	3 (2.0)	3 (3.7)	1 (1.2)
Decreased appetite	19 (12.8)	0 (0.0)	6 (7.4)	0 (0.0)
Decreased appetite	17 (11.5)	2 (1.4)	4 (4.9)	0 (0.0)
Upper respiratory tract infection	16 (10.8)	1 (0.7)	5 (6.2)	2 (2.5)
Dizziness	13 (8.8)	0 (0.0)	10 (12.3)	0 (0.0)
Rash	16 (10.8)	0 (0.0)	6 (7.4)	1 (1.2)

Notes: TEAE, treatment-emergent adverse event; SAE, serious adverse event. Percentages use each RIME group denominator in the chemotherapy plus PD-1 inhibitor group. Only patients with available RIME measurements were included. RIME grouping was based on logpoly_RNA_ave using the exploratory cutoff of 2.23 derived by maximally selected rank statistics based on PFS time and event status.
